# Generation and evaluation of synthetic patient data

**DOI:** 10.1186/s12874-020-00977-1

**Published:** 2020-05-07

**Authors:** Andre Goncalves, Priyadip Ray, Braden Soper, Jennifer Stevens, Linda Coyle, Ana Paula Sales

**Affiliations:** 1 Lawrence Livermore National Laboratory, 7000 East Ave, Livermore, CA USA; 2 Information Management Systems, 1455 Research Blvd, Suite 315, Rockville, MD USA

**Keywords:** Synthetic data generation, Cancer patient data, Information disclosure, Generative models

## Abstract

**Background:**

Machine learning (ML) has made a significant impact in medicine and cancer research; however, its impact in these areas has been undeniably slower and more limited than in other application domains. A major reason for this has been the lack of availability of patient data to the broader ML research community, in large part due to patient privacy protection concerns. High-quality, realistic, synthetic datasets can be leveraged to accelerate methodological developments in medicine. By and large, medical data is high dimensional and often categorical. These characteristics pose multiple modeling challenges.

**Methods:**

In this paper, we evaluate three classes of synthetic data generation approaches; probabilistic models, classification-based imputation models, and generative adversarial neural networks. Metrics for evaluating the quality of the generated synthetic datasets are presented and discussed.

**Results:**

While the results and discussions are broadly applicable to medical data, for demonstration purposes we generate synthetic datasets for cancer based on the publicly available cancer registry data from the Surveillance Epidemiology and End Results (SEER) program. Specifically, our cohort consists of breast, respiratory, and non-solid cancer cases diagnosed between 2010 and 2015, which includes over 360,000 individual cases.

**Conclusions:**

We discuss the trade-offs of the different methods and metrics, providing guidance on considerations for the generation and usage of medical synthetic data.

## Background

Increasingly, large amounts and types of patient data are being electronically collected by healthcare providers, governments, and private industry. While such datasets are potentially highly valuable resources for scientists, they are generally not accessible to the broader research community due to patient privacy concerns. Even when it is possible for a researcher to gain access to such data, ensuring proper data usage and protection is a lengthy process with strict legal requirements. This can severely delay the pace of research and, consequently, its translational benefits to patient care.

To make sensitive patient data available to others, data owners typically de-identify or anonymize the data in a number of ways, including removing identifiable features (e.g., names and addresses), perturbing them (e.g., adding noise to birth dates), or grouping variables into broader categories to ensure more than one individual in each category [1]. While the residual information contained in properly anonymized data alone may not be used to re-identify individuals, once linked to other datasets (e.g., social media platforms), they may contain enough information to identify specific individuals. Efforts to determine the efficacy of de-identification methods have been inconclusive, particularly in the context of large datasets [2]. As such, it remains extremely difficult to guarantee that re-identification of individual patients is not a possibility with current approaches.

Given the risks of re-identification of patient data and the delays inherent in making such data more widely available, synthetically generated data is a promising alternative or addition to standard anonymization procedures. Synthetic data generation has been researched for nearly three decades [3] and applied across a variety of domains [4, 5], including patient data [6] and electronic health records (EHR) [7, 8]. It can be a valuable tool when real data is expensive, scarce or simply unavailable. While in some applications it may not be possible, or advisable, to derive new knowledge directly from synthetic data, it can nevertheless be leveraged for a variety of secondary uses, such as educative or training purposes, software testing, and machine learning and statistical model development. Depending on one’s objective, synthetic data can either entirely replace real data, augment it, or be used as a reasonable proxy to accelerate research.

A number of synthetic patient data generation methods aim to minimize the use of actual patient data by combining simulation, public population-level statistics, and domain expert knowledge bases [7–10]. For example, in Dube and Gallagher [8] synthetic electronic health records are generated by leveraging publicly available health statistics, clinical practice guidelines, and medical coding and terminology standards. In a related approach, patient demographics (obtained from actual patient data) are combined with expert-curated, publicly available patient care patterns to generate synthetic electronic medical records [9]. While the emphasis on not accessing real patient data eliminates the issue of re-identification, this comes at the cost of a heavy reliance on domain-specific knowledge bases and manual curation. As such, these methods may not be readily deployable to new cohorts or sets of diseases. Entirely data-driven methods, in contrast, produce synthetic data by using patient data to learn parameters of generative models. Because there is no reliance on external information beyond the actual data of interest, these methods are generally disease or cohort agnostic, making them more readily transferable to new scenarios.

Synthetic patient data has the potential to have a real impact in patient care by enabling research on model development to move at a quicker pace. While there exists a wealth of methods for generating synthetic data, each of them uses different datasets and often different evaluation metrics. This makes a direct comparison of synthetic data generation methods surprisingly difficult. In this context, we find that there is a void in terms of guidelines or even discussions on how to compare and evaluate different methods in order to select the most appropriate one for a given application. Here, we have conducted a systematic study of several methods for generating synthetic patient data under different evaluation criteria. Each metric we use addresses one of three criteria of high-quality synthetic data: 1) Fidelity at the individual sample level (e.g., synthetic data should not include prostate cancer in a female patient), 2) Fidelity at the population level (e.g., marginal and joint distributions of features), and 3) privacy disclosure. The scope of the study is restricted to data-driven methods only, which, as per the above discussion, do not require manual curation or expert-knowledge and hence can be more readily deployed to new applications. While there is no single approach for generating synthetic data which is the best for all applications, or even a one-size-fits-all approach to evaluating synthetic data quality, we hope that the current discussion proves useful in guiding future researchers in identifying appropriate methodologies for their particular needs.

The paper is structured as follows. We start by providing a focused discussion on the relevant literature on data-driven methods for generation of synthetic data, specifically on categorical features, which is typical in medical data and presents a set of specific modeling challenges. Next, we describe the methods compared in the current study, along with a brief discussion of the advantages and drawbacks of each approach. We then describe the evaluation metrics, providing some intuition on the utility and limitation of each. The datasets used and our experimental setup are presented. Finally, we discuss our results followed by concluding remarks.

### Related work

Synthetic data generation can roughly be categorized into two distinct classes: process-driven methods and data-driven methods. Process-driven methods derive synthetic data from computational or mathematical models of an underlying physical process. Examples include numerical simulations, Monte Carlo simulations, agent-based modeling, and discrete-event simulations. Data-driven methods, on the other hand, derive synthetic data from generative models that have been trained on observed data. Because this paper is mainly concerned with data-driven methods, we briefly review the state-of-the-art methods in this class of synthetic data generation techniques. We consider three main types of data-driven methods: Imputation based methods, full joint probability distribution methods, and function approximation methods.

Imputation based methods for synthetic data generation were first introduced by Rubin [3] and Little [11] in the context of Statistical Disclosure Control (SDC), or Statistical Disclosure Limitation (SDL) [4]. SDC and SDL methodologies are primarily concerned with reducing the risk of disclosing sensitive data when performing statistical analyses. A general survey paper on data privacy methods related to SDL is Matthews and Harel [12]. Standard techniques are based on multiple imputation [13], treating sensitive data as missing data and then releasing randomly sampled imputed values in place of the sensitive data. These methods were later extended to the fully synthetic case by Raghunathan, Reiter and Rubin [14]. Early methods focused on continuous data with extensions to categorical data following [15]. Generalized linear regression models are typically used, but non-linear methods (such as Random Forest and neural networks) can and have been used [16]. Remedies for some of the shortcomings with multiple imputation for generating synthetic data are offered in Loong and Rubin [17]. An empirical study of releasing synthetic data under the methods proposed in Raghunathan, Reiter and Rubin [14] is presented in Reiter and Drechsler [18]. Most of the SDC/SDL literature focuses on survey data from the social sciences and demography. The generation of synthetic electronic health records has been addressed in Dube and Gallagher [8].

Multiple imputation has been the de facto method for generating synthetic data in the context of SDC and SDL. While imputation based methods are fully probabilistic, there is no guarantee that the resulting generative model is an estimate of the full joint probability distribution of the sampled population. In some applications, it may be of interest to model this probability distribution directly, for example if parameter interpretability is important. In this case, any statistical modeling procedure that learns a joint probability distribution is capable of generating fully synthetic data.

In the case of generating synthetic electronic health care records, one must be able to handle multivariate categorical data. This is a challenging problem, particularly in high dimensions. It is often necessary to impose some sort of dependence structure on the data [19]. For example, Bayesian networks, which approximate a joint distribution using a first-order dependence tree, have been proposed in Zhang et al. [20] as a method for generating synthetic data with privacy constraints. More flexible non-parametric methods need not impose such dependence structures on the distributions. Examples of Bayesian non-parametric methods for multidimensional categorical data include latent Gaussian process methods [21] and Dirichlet mixture models [22].

Synthetic data has recently attracted attention from the machine learning (ML) and data science communities for reasons other than data privacy. Many state-of-the-art ML algorithms are based on function approximation methods such as deep neural networks (DNN). These models typically have a large number of parameters and require large amounts of data to train. When labeled data sets are impossible or expensive to obtain, it has been proposed that synthetically generated training data can complement scarce real data [23]. Similarly, transfer learning from synthetic data to real data to improve ML algorithms has also been explored [24, 25]. Thus data augmentation methods from the ML literature are a class of synthetic data generation techniques that can be used in the bio-medical domain.

Generative Adversarial Networks (GANs) are a popular class of DNNs for unsupervised learning tasks [26]. In particular, they produce two jointly-trained networks; one which generates synthetic data intended to be similar to the training data, and one which tries to discriminate the synthetic data from the true training data. They have proven to be very adept at learning high-dimensional, continuous data such as images [26, 27]. More recently GANs for categorical data have been proposed in Camino, Hammerschmidt and State [28] with specific applications to synthetic EHR data in Choi et al. [29].

Finally, we note that several open-source software packages exist for synthetic data generation. Recent examples include the R packages *synthpop* [30] and *SimPop* [31], the Python package *DataSynthesizer* [5], and the Java-based simulator *Synthea* [7].

## Methods

### Methods for synthetic data generation

In this paper we investigate various techniques for synthetic data generation. The techniques we investigate range from fully generative Bayesian models to neural network based adversarial models. We next provide brief descriptions of the synthetic data generation approaches considered.

#### Sampling from independent marginals

The Independent marginals (IM) method is based on sampling from the empirical marginal distributions of each variable. The empirical marginal distribution is estimated from the observed data. We next summarize the key advantages (+) and disadvantages (-) of this approach.

- This approach is computationally efficient and the estimation of marginal distributions for different variables may be done in parallel.
- IM does not capture statistical dependencies across variables, and hence the generated synthetic data may fail to capture the underlying structure of the data.

This method is included in our analysis solely as a simple baseline for other more complex approaches.

#### Bayesian network

Bayesian networks (BN) are probabilistic graphical models where each node represents a random variable, while the edges between the nodes represent probabilistic dependencies among the corresponding random variables. For synthetic data generation using a Bayesian network, the graph structure and the conditional probability distributions are inferred from the real data. In BN, the full joint distribution is factorized as:

1 $$ p(\mathbf{x}) = \prod_{v \in V}p(x_{v}|\mathbf{x}_{\text{pa}(v)})   $$

where *V* is the set of random variables representing the categorical variables and **x**_pa(*v*)_ is the subset of parent variables of *v*, which is encoded in the directed acyclic graph.

The learning process consists of two steps: (*i*) learning a directed acyclic graph from the data, which expresses all the pairwise conditional (in)dependence among the variables, and (*ii*) estimating the conditional probability tables (CDP) for each variable via maximum likelihood. For the first step we use the Chow-Liu tree [19] method, which seeks a first-order dependency tree-based approximation with the smallest KL-divergence to the actual full joint probability distribution. The Chow-Liu algorithm provides an approximation and cannot represent higher-order dependencies. Nevertheless, it has been shown to provide good results for a wide range of practical problems.

The graph structure inferred from the real data encodes the conditional dependence among the variables. In addition, the inferred graph provides a visual representation of the variables’ relationships. Synthetic data may be generated by sampling from the inferred Bayesian network. We next summarize the key advantages and disadvantages of this approach.

- BN is computationally efficient and scales well with the dimensionality of the dataset.
- The directed acyclic graph can also be utilized for exploring the causal relationships across the variables.
- Even though the full joint distribution’s factorization, as given by Eq. (1), is general enough to include any possible dependency structure, in practice, simplifying assumptions on the graphical structure are made to ease model inference. These assumptions may fail to represent higher-order dependencies.
- The inference approach adopted in this paper is applicable only to discrete data. In addition, the Chow-Liu heuristic used here constructs the directed acyclic graph in a greedy manner. Therefore, an optimal first-order dependency tree is not guaranteed.

#### Mixture of product of multinomials

Any multivariate categorical data distribution can be expressed as a mixture of product of multinomials (MPoM) [22],

2 $$  p(x_{i1}=c_{1}, \ldots, x_{ip}=c_{p}) = \sum_{h=1}^{k}\nu_{h}\prod_{j=1}^{p}\psi_{hc_{j}}^{(j)}  $$

where *x*_*i*_=(*x*_*i*1_,…,*x*_*ip*_) represents a vector of *p* categorical variables, *k* is the number of mixture components, *ν*_*h*_ is the weight associated with the *h*-th mixture component, and $\psi _{hc_{j}}^{(j)} = Pr(x_{ij}= c_{j}|z_{i} = h)$ is the probability of *x*_*ij*_=*c*_*j*_ given allocation of individual *i* to cluster *h*, where *z*_*i*_ is a cluster indicator. Although any multivariate distribution may be expressed as in (2) for a sufficiently large *k*, proper choice of *k* is troublesome. To obtain *k* in a data-driven manner, Dunson and Xing [22] proposed a Dirichlet process mixture of product multinomials to model high-dimensional multivariate categorical data. We next summarize the key advantages and disadvantages of this approach.

- Theoretical guarantees exist regarding the flexibility of mixture of product multinomials to model any multivariate categorical data.
- The Dirichlet process mixture of product of multinomials is a fully conjugate model and efficient inference may be done via a Gibbs sampler.
- Sampling based inference can be very slow in high dimensional problems.
- While extending the model to mixed data types (such as continuous and categorical) is relatively straightforward, theoretical guarantees do not exist for mixed data types.

#### Categorical latent Gaussian process

The categorical latent Gaussian process (CLGP) is a generative model for multivariate categorical data [21]. CLGP uses a lower dimensional continuous latent space and non-linear transformations for mapping the points in the latent space to probabilities (via softmax) for generating categorical values. The authors employ standard Normal priors on the latent space and sparse Gaussian process (GPs) mappings to transform the latent space. For modeling clinical data related to cancer, the model assumes that each patient record (a data vector containing a set of categorical variables) has a continuous latent low-dimensional representation. The proposed model is not fully conjugate, but model inference may be performed via variational techniques.

The hierarchical CLGP model [21] is provided below:

$$\begin{array}{*{20}l} x_{nq} & \stackrel{iid}{\sim} \mathcal{N}\left(0, \sigma^{2}_{x}\right)\\ \mathcal{F}_{dk} & \stackrel{iid}{\sim} \mathcal{GP}(0, \mathbf{K}_{d})\\ f_{ndk} & = \mathcal{F}_{dk}(\mathbf{x}_{n}), \;\;u_{mdk} = \mathcal{F}_{dk}(\mathbf{z}_{m})\\ y_{nd} & \sim \text{Softmax}(\mathbf{f}_{nd}) \end{array} $$

for *n*∈[*N*] (the set of naturals between 1 and N), *q*∈[*Q*], *d*∈[*D*], *k*∈[*K*], *m*∈[*M*], covariance matrices **K**_*d*_, and where the Softmax distribution is defined as,

3 $$ \begin{aligned}\text{Softmax}(y=k;\mathbf{f}) & = \text{Categorical}\left(\frac{\text{exp}(f_{k})}{\text{exp}(\text{lse}(\mathbf{f}))}\right),\\ \text{lse}(\mathbf{f}) & = \log \left(1 + \sum_{k'=1}^{K}\text{exp}(f_{k'})\right) \end{aligned} $$

for *k*=0,...,*K* and with *f*_0_:=0. Each patient is represented in the latent space as **x**_*n*_. For each feature *d*, **x**_*n*_ has a sequence of weights (*f*_*nd*1_,...,*f*_*ndK*_), corresponding to each possible feature level *k*, that follows a Gaussian process. Softmax returns a feature value *y*_*nd*_ based on these weights, resulting in the patient’s feature vector **y**_*n*_=(*y*_*n*1_,...,*y*_*nD*_). Note that CLGP does not explicitly model dependence across variables (features). However, the Gaussian process explicitly captures the dependence across patients and the shared low-dimensional latent space implicitly captures dependence across variables.

We next summarize the key advantages and disadvantages of this approach.

- Like BN and MPoM, CLGP is a fully generative Bayesian model, but has richer latent non-linear mappings that allows for representation of very complex full joint distributions.
- The inferred low-dimensional latent space in CLGP may be useful for data visualization and clustering.
- Inference for CLGP is considerably more complex than other models due to its non-conjugacy. An approximate Bayesian inference method such as variational Bayes (VB) is required.
- VB for CLGP requires several other approximations such as low-rank approximation for GPs as well as Monte Carlo integration. Hence, the inference for CLGP scales poorly with data size.

#### Generative adversarial networks

Generative adversarial networks (GANs) [26] have recently been shown to be remarkably successful for generating complex synthetic data, such as images and text [32–34]. In this approach, two neural networks are trained jointly in a competitive manner: the first network tries to generate realistic synthetic data, while the second one attempts to discriminate real and synthetic data generated by the first network. During the training each network pushes the other to perform better. A widely known limitation of GANs is that it is not directly applicable for generating categorical synthetic datasets, as it is not possible to compute the gradients on latent categorical variables that are required for training via backpropagation. As clinical patient data are often largely categorical, recent works like medGAN [29] have applied autoencoders to transform categorical data to a continuous space, after which GANs can be applied for generating synthetic electronic health records (EHR). However, medGAN is applicable to binary and count data, and not multi-categorical data. In this paper we adopt the multi-categorical extension of medGAN, called MC-MedGAN [28] to generate synthetic data related to cancer. We next summarize the key advantages and disadvantages of this approach.

- Unlike POM, BN and CLGP, MC-MedGAN is a generative approach which does not require strict probabilistic model assumptions. Hence, it is more flexible compared to BN, CLGP and POM.
- GANs-based models can be easily extended to deal with mixed data types, e.g., continuous and categorical variables.
- MC-MedGAN is a deep model and has a very large number of parameters. Proper choice of multiple tuning parameters (hyper-parameters) is difficult and time consuming.
- GANs are known to be difficult to train as the process of solving the associated min-max optimization problem can be very unstable. However, recently proposed variations of GAN such as Wasserstein GANs, and its variants, have significantly alleviated the problem of stability of training GANs [35, 36].

#### Multiple imputation

Multiple imputation based methods have been very popular in the context of synthetic data generation, especially for applications where a part of the data is considered sensitive [4]. Among the existing imputation methods, the Multivariate Imputation by Chained Equations (MICE) [37] has emerged as a principled method for masking sensitive content in datasets with privacy constraints. The key idea is to treat sensitive data as missing data. One then imputes this “missing” data with randomly sampled values generated from models trained on the nonsensitive variables.

As discussed earlier, generating fully synthetic data often utilizes a generative model trained on an entire dataset. It is then possible to generate complete synthetic datasets from the trained model. This approach differs from standard multiple imputation methods such as MICE, which train on subsets of nonsensitive data to generate synthetic subsets of sensitive data. In this paper we use a variation of MICE for the task of fully synthetic data generation. Model inference proceeds as follows.

- Define a topological ordering of the variables.
- Compute the empirical marginal probability distribution for the first variable.
- For each successive variables in the topological order, learn a probabilistic model for the conditional probability distribution on the current variable given the previous variables, that is, *p*(*x*_*v*_|**x**_:*v*_), which is done by regressing the *v*-th variable on all its predecessors as independent variables.

In the sampling phase, the first variable is sampled from the empirical distribution and the remaining variables are randomly sampled from the inferred conditional distributions following the topological ordering. While modeling the conditional distributions with generalized linear models is very popular, other non-linear techniques such as random forests and neural nets may be easily integrated in this framework.

For the MICE variation used here, the full joint probability distribution is factorized as follows:

4 $$ p(\mathbf{x}) = \prod_{v \in V} p(x_{v}|\mathbf{x}_{:v})  $$

where *V* is the set of random variables representing the variables to be generated, and *p*(*x*_*v*_|**x**_:*v*_) is the conditional probability distribution of the *v*-th random variable given all its predecessors. Clearly, the definition of the topological ordering plays a crucial role in the model construction. A common approach is to sort the variables by the number of levels either in ascending or descending order.

We next summarize the key advantages and disadvantages of this approach.

- MICE is computationally fast and can scale to very large datasets, both in the number of variables and samples.
- It can easily deal with continuous and categorical values by properly choosing either a Softmax or a Gaussian model for the conditional probability distribution for a given variable.
- While MICE is probabilistic, there is no guarantee that the resulting generative model is a good estimate of the underlying joint distribution of the data.
- MICE strongly relies on the flexibility of the model for the conditional probability distributions and also the topological ordering of the directed acyclic graph.

### Evaluation metrics

To measure the quality of the synthetic data generators, we use a set of complementary metrics that can be divided into two groups: (*i*) data utility, and (*ii*) information disclosure. In the former, the metrics gauge the extent to which the statistical properties of the real (private) data are captured and transferred to the synthetic dataset. In the latter group, the metrics measure how much of the real data may be revealed (directly or indirectly) by the synthetic data. It has been well documented that increased generalization and suppression in anonymized data (or smoothing in synthetic data) for increased privacy protection can lead to a direct reduction in data utility [38]. In the context of this trade-off between data utility and privacy, evaluation of models for generating such data must take both opposing facets of synthetic data into consideration.

#### Data utility metrics

In this group, we consider the following metrics: *Kullback-Leibler (KL) divergence*, *pairwise correlation difference*, *log-cluster*, *support coverage*, and *cross-classification*.

The **Kullback-Leibler (KL) divergence** is computed over a pair of real and synthetic marginal probability mass functions (PMF) for a given variable, and it measures the similarity of the two PMFs. When both distributions are identical, the KL divergence is zero, while larger values of the KL divergence indicate a larger discrepancy between the two PMFs. Note that the KL divergence is computed for each variable independently; therefore, it does not measure dependencies among the variables. The KL divergence of two PMFs, *P*_*v*_ and *Q*_*v*_ for a given variable *v*, is computed as follows:

5 $$ D_{\text{KL}}(P_{v}\|Q_{v}) = \sum_{i=1}^{|v|}P_{v}(i)\log \frac{P_{v}(i)}{Q_{v}(i)},  $$

where |*v*| is the cardinality (number of levels) of the categorical variable *v*. Note that the KL divergence is defined at the variable level, not over the entire dataset.

The **pairwise correlation difference (PCD)** is intended to measure how much correlation among the variables the different methods were able to capture. PCD is defined as:

6 $$ PCD(X_{R}, X_{S}) = \|Corr(X_{R}) - Corr(X_{S})\|_{F},  $$

where *X*_*R*_ and *X*_*S*_ are the real and synthetic data matrices, respectively. PCD measures the difference in terms of Frobennius norm of the Pearson correlation matrices computed from real and synthetic datasets. The smaller the PCD, the closer the synthetic data is to the real data in terms of linear correlations across the variables. PCD is defined at the dataset level.

The **log-cluster** metric [39] is a measure of the similarity of the underlying latent structure of the real and synthetic datasets in terms of clustering. To compute this metric, first, the real and synthetic datasets are merged into one single dataset. Second, we perform a cluster analysis on the merged dataset with a fixed number of clusters G using the *k*-means algorithm. Finally, we calculate the metric as follows:

7 $$ U_{c}(X_{R}, X_{S}) = \log\left(\frac{1}{G}\sum_{j=1}^{G} \left[\frac{n_{j}^{R}}{n_{j}} - c\right]^{2}\right),  $$

where *n*_*j*_ is the number of samples in the *j*-th cluster, $n_{j}^{R}$ is the number of samples from the real dataset in the *j*-th cluster, and *c*=*n*^*R*^/(*n*^*R*^+*n*^*S*^). Large values of *U*_*c*_ indicate disparities in the cluster memberships, suggesting differences in the distribution of real and synthetic data. In our experiments, the number of clusters was set to 20. The *log-cluster* metric is defined at the dataset level.

The **support coverage** metric measures how much of the variables support in the real data is covered in the synthetic data. The metric considers the ratio of the cardinalities of a variable’s support (number of levels) in the real and synthetic data. Mathematically, the metric is defined as the average of such ratios over all variables:

8 $$ S_{c}(X_{R}, X_{S}) = \frac{1}{V}\sum_{v=1}^{V} \frac{|\mathcal{S}^{v}|}{|\mathcal{R}^{v}|}  $$

where $\mathcal {R}^{v}$ and $\mathcal {S}^{v}$ are the support of the *v*-th variable in the real and synthetic data, respectively. At its maximum (in the case of perfect support coverage), this metric is equal to 1. This metric penalizes synthetic datasets if less frequent categories are not well represented. It is defined at the dataset level.

The **cross-classification** metric is another measure of how well a synthetic dataset captures the statistical dependence structures existing in the real data. Unlike PCD, in which statistical dependence is measured by Pearson correlation, cross-classification measures dependence via predictions generated for one variable based on the other variables (via a classifier).

We consider two cross-classification metrics in this paper. The first cross-classification metric, referred to as CrCl-RS, involves training on the real data and testing on hold-out data from both the real and synthetic datasets. This metric is particularly useful for evaluating if the statistical properties of the real data are similar to those of the synthetic data. The second cross-classification metric, referred to as (CrCl-SR), involves training on the synthetic data and testing on hold-out data from both real and synthetic data. This metric is particularly useful in determining if scientific conclusions drawn from statistical/machine learning models trained on synthetic datasets can safely be applied to real datasets. We next provide additional details regarding the cross-classification metric CrCl-RS. The cross-classification metric CrCl-SR is computed in a similar manner.

**Fig. 1:**
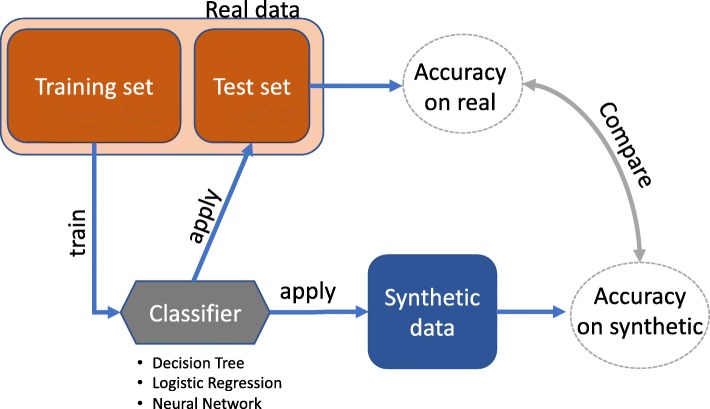
Schematic view of the *cross-classification* metric computation. It consists of the following steps: (1) real data is split into training and test sets; (2) classifier is trained on the training set; (3) classifier is applied on both test set (real) and synthetic data; and (4) the ratio of the classification performances is calculated

The available real data is split into training and test sets. A classifier is trained on the *training set* (real) and applied to both *test set* (hold out real) and the *synthetic data*. Classification performance metrics are computed on both sets. CrCl-RS is defined as the ratio between the performance on synthetic data and on the held out real data. Figure 1 presents a schematic representation of the *cross classification* computation. Clearly, the classification performance is dependent on the chosen classifier. Here, we consider a decision tree as the classifier due to the discrete nature of the dataset. To perform the classification, one of the variables is used as a target, while the remaining are used as predictors. This procedure is repeated for each variable as target, and the average value is reported. In general, for both cross-classification metrics, a value close to 1 is ideal.

#### Disclosure metrics

There are two broad classes of privacy disclosure risks: *identity disclosure* and *attribute disclosure*. Identity or membership disclosure refers to the risk of an intruder correctly identifying an individual as being included in the confidential dataset. This attack is possible when the attacker has access to a complete set of patient records. In the fully synthetic case, the attacker wants to know whether a private record the attacker has access to was used for training the generative model that produced the publicly available synthetic data. Attribute disclosure refers to the risk of an intruder correctly guessing the original value of the synthesized attributes of an individual whose information is contained in the confidential dataset. In the “Experimental analysis on SEER’s research dataset” section, we will show results for both privacy disclosure metrics. Next, we provide details on how these metrics are computed.

In **membership disclosure** [29], one claims that a patient record *x* was present in the training set if there is at least one synthetic data sample within a certain distance (for example, in this paper we have considered Hamming distance) to the record *x*. Otherwise, it is claimed not to be present in the training set. To compute the membership disclosure of a given method *m*, we select a set of *r* patient records used to train the generative model and another set of *r* patient records that were not used for training, referred to as *test records*. With the possession of these 2*r* patient records and a synthetic dataset generated by the method *m*, we compute the claim outcome for each patient record by calculating its Hamming distance to each sample from the synthetic dataset, and then determining if there is a synthetic data sample within a prescribed Hamming distance. For each claim outcome there are four possible scenarios: true positive (attacker correctly claims their targeted record is in the training set), false positive (attacker incorrectly claims their targeted record is in the training set), true negative (attacker correctly claims their targeted record is not in the training set), or false negative (attacker incorrectly claims their targeted record is not in the training set). Finally, we compute the precision and recall of the above claim outcomes. In our experiments, we set *r*=1000 records and used the entire set of synthetic data available.

**Attribute disclosure** [29] refers to the risk of an attacker correctly inferring sensitive attributes of a patient record (e.g., results of medical tests, medications, and diagnoses) based on a subset of attributes known to the attacker. For example, in the fully synthetic data case, an attacker can first extract the *k* nearest neighboring patient records of the synthetic dataset based on the known attributes, and then infer the unknown attributes via a majority voting rule. The chance of unveiling the private information is expected to be low if the synthetic generation method has not memorized the private dataset. The number of known attributes, the size of the synthetic dataset, and the number of *k* nearest neighbors used by the attacker affect the chance of revealing the unknown attributes. In our experiments we investigate the chance that an attacker can reveal all the unknown attributes, given different numbers of known attributes and several choices of *k*.

In addition to membership and attribute attacks, the framework of differential privacy has garnered a lot of interest [40–42]. The key idea is to protect the information of every individual in the database against an adversary with complete knowledge of the rest of the dataset. This is achieved by ensuring that the synthetic data does not depend too much on the information from any one individual. A significant amount of research has been devoted on designing *α*-differential or (*α*,*δ*)-differential algorithms [43, 44]. An interesting direction of research has been in converting popular machine learning algorithms, such as deep learning algorithms, to differentially private algorithms via techniques such as gradient clipping and noise addition [45, 46]. In this paper, we have not considered differential privacy as a metric. While the algorithms discussed in this paper such as MC-MedGAN or MPoM may be modified to introduce differential privacy, that is beyond the scope of this paper.

### Experimental analysis on SEER’s research dataset

In this section we describe the data used in our experimental analysis. We considered the methods previously discussed, namely Independent Marginals (IM), Bayesian Network (BN), Mixture of Product of Multinomials (MPoM), CLGP, MC-MedGAN, and MICE. Three variants of MICE were considered: MICE with Logistic Regression (LR) as classifier and variables ordered by the number of categories in an ascending manner (MICE-LR), MICE with LR and ordered in a descending manner (MICE-LR-DESC), and MICE with Decision Tree as classifier (MICE-DT) in ascending order. MICE-DT with descending and ascending order produced similar results and only one is reported in this paper for brevity.

#### Dataset variable selection

A subset of variables from the public research SEER’s dataset^1^ was used in this experiment. The variables were selected after taking into account the characteristics of the variables and their temporal availability, as some variables were more recently introduced as compared to others. Two sets of variables were created: (*i*) a set of 8 variables with a small number of categories (small-set); and (*ii*) a larger set with ∼40 variables (large-set) that includes variables with a large number (hundreds) of categories. We want to see the relative performances of the different synthetic data generation approaches on a relatively easy dataset (small-set) and on a more challenging dataset (large-set).

The SEER’s research dataset is composed of sub-datasets, where each sub-dataset contains diagnosed cases of a specific cancer type collected from 1973 to 2015. For this analysis we considered the sub-datasets from patients diagnosed with *breast* cancer (BREAST), *respiratory* cancer (RESPIR), and *lymphoma of all sites and leukemia* (LYMYLEUK). We used data from cases diagnosed between 2010 and 2015 due to the nonexistence of some of variables prior to this period. The number of patient records in the BREAST, RESPIR, and LYMYLEUK datasets are 169,801; 112,698; and 84,132; respectively. We analyze the performance of the methods on each dataset separately. Table 1 presents the variables selected. A pre-processing step in some cases involves splitting a more complex variable into two variables, as some variables originally contained both categorical and integer (count) values.

**Table 1:** Two sets of variables from SEER’s research dataset

Feature set	Variables
small-set	AGE_DX, BEHO3V, DX_CONF, GRADE, LATERAL, PRIMSITE ^∗^, SEQ_NUM, SEX
large-set	AGE_DX, BEHO3V, CS1SITE, CS2SITE, CS3SITE, CS4SITE ^*†*^, CS5SITE ^*†*^, CS6SITE ^*†*^, CS7SITE ^*†*∘^, CS15SITE ^*†*∘^, CSEXTEN, CSLYMPHN, CSMETSDX, CSMETSDXBR_PUB, CSMETSDXB_PUB, CSMETSDXLIV_PUB, CSMETSDXLUNG_PUB, CSMTEVAL, CSRGEVAL, CSTSEVAL, CSVCURRENT, CSVFIRST, DX_CONF, GRADE, HISTO3V, LATERAL, MAR_STAT, NHIADE, NO_SURG, PRIMSITE, RACE1V, REC_NO, REG, REPT_SRC, SEQ_NUM, SEX, SURGSITF, TYPE_FU, YEAR_DX, YR_BRTH

small-set contains variables with low levels; while large-set contains a large number of variables including a few with large number of levels. ^∗^variable PRIMSITE is only considered for BREAST dataset as it has a large number of levels for LYMYLEUK and RESPIR. ^*†*^ indicates that the variable is not present in the LYMYLEUK dataset. ^∘^ indicates that the variable is not present in the RESPIR dataset

The number of levels (categories) in each variable is diverse. In the small-set feature set the number of categories ranges from 1 to 14, while for the large-set it ranges from 1 to 257. The number of levels for each variable in presented Tables 2 and 3.

**Table 2:** Number of levels in each categorical variable for the feature set small-set

	AGE_DX	BEHO3V	DX_CONF	GRADE	LATERAL	PRIMSITE	SEQ_NUM	SEX
BREAST	11	2	9	5	5	9	10	2
LYMYLEUK	12	1	9	5	7	-	14	2
RESPIR	12	2	8	5	6	-	11	2

**Table 3:** Number of levels in each categorical variable in the feature set large-set

	BREAST	LYMYLEUK	RESPIR
AGE_DX	11	12	12
BEHO3V	2	1	2
CS15SITE	7	-	-
CS1SITE	7	12	100
CS2SITE	7	6	8
CS3SITE	56	13	10
CS4SITE	5	-	10
CS5SITE	4	-	8
CS6SITE	8	-	9
CS7SITE	12	-	-
CSEXTEN	37	39	91
CSLYMPHN	36	21	23
CSMETSDX	9	12	31
CSMETSDXBR_PUB	4	4	4
CSMETSDXB_PUB	4	4	4
CSMETSDXLIV_PUB	4	4	4
CSMETSDXLUNG_PUB	4	4	4
CSMTEVAL	8	4	8
CSRGEVAL	8	6	8
CSTSEVAL	8	8	8
CSVCURRENT	5	5	5
CSVFIRST	10	11	10
DX_CONF	9	9	8
GRADE	5	5	5
HISTO3V	102	100	169
LATERAL	5	7	6
MAR_STAT	7	7	7
NHIADE	9	9	9
NO_SURG	8	8	8
PRIMSITE	9	257	29
RACE1V	29	28	30
REC_NO	7	11	9
REG	9	9	9
REPT_SRC	8	8	8
SEQ_NUM	10	14	11
SEX	2	2	2
SURGSITF	7	7	7
TYPE_FU	2	2	2
YEAR_DX	6	6	6
YR_BRTH	96	111	112

Dash ’-’ indicates that the variable (row) was not considered for that dataset (column) as the variable is not existent for that cancer type

**Fig. 2:**
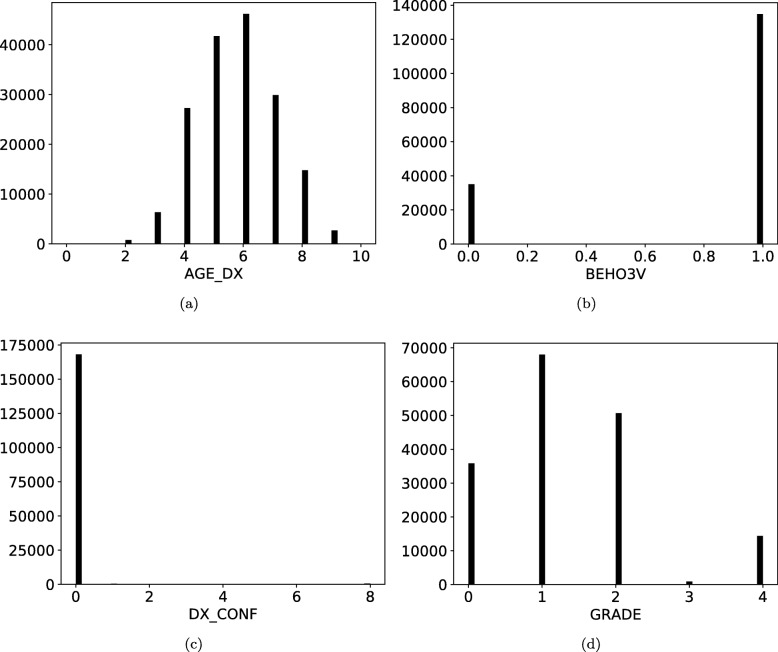
Histogram of four BREAST small-set variables from the real dataset. Levels’ distributions are clearly imbalanced

Figure 2 depicts the histogram of some variables in the BREAST small-set dataset. Noticeably, the levels’ distributions are imbalanced and many levels are underrepresented in the real dataset. For example, variable DX_CONF mostly contains records with the same level, and LATERAL only has records with 2 out of 5 possible levels. This imbalance may inadvertently lead to disclosure of information in the synthetic dataset, as the methods are more prone to overfit when the data has a smaller number of possible record configurations.

#### Implementation details and hyper-parameter selection

When available we used the code developed by the authors of the paper proposing the synthetic data generation method. For CLGP, we used the code from the authors’ GitHub repository [47]. For MC-MedGAN, we also utilized the code from the authors [48]. For the Bayesian networks, we used two Python packages: *pomegranate* [49] and *libpgm* [50]. All other methods were implemented by ourselves. The hyper-parameter values used for all methods were selected via grid-search. The selected values were those which provided the best performance for the **log-cluster** utility metric. This metric was used as it is the only metric, in our pool of utility metrics, that measures the similarity of the full real and synthetic data distributions, and not only the marginal distributions or only the relationship across variables. The range of hyper-parameter values explored for all methods is described below.

### Hyper-parameter values

To select the best hyper-parameter values for each method, we performed a grid-search over a set of candidate values. Below we present the set of values tested. Bayesian networks and Independent Marginals did not have hyper-parameters to be selected. MPoM: The truncated Dirichlet process prior uses 30 clusters (*k*=30), concentration parameter *α*=10, and 10,000 Gibbs sampling steps with 1,000 burn-in steps, for both small-set and large-set. For the grid-search selection, we tested *k*=[5,10,20,30,50], and *k*=30 led to the best log-cluster performance. CLGP: We used 100 inducing points and 5-dimensional latent space for small-set; and 100 inducing points and 10-dimensional latent space for the large-set. Increasing the number of inducing points usually leads to a better utility performance, but the computational cost increases substantially. Empirically, we found that 100 inducing points provides an adequate balance between utility performance and computational cost. For the small-set, the values tested for the latent space size was [2, 3, 4, 5, 6] dimensions; and for the large-set [5, 10, 15] dimensions. MC-MedGAN: We tested two variations of model configuration used by the authors in the original paper. The variations were a smaller model (Model 1) and a bigger model (Model 2), in terms of number of parameters (See Table 4). The bigger model is more flexible and in theory can capture highly non-linear relations among the attributes, and provide better continuous representation of the discrete data, via an autoencoder. On the other hand, the smaller model is less prone to overfit the private data. We also noticed that a proper definition for the learning rate, both the pre-trained autoenconder and GAN, is crucial for the model performance. We tested both models with learning rate of [1e-2, 1e-3, 1e-4]. We then selected the best performing model for each feature set considering the log-cluster utility metric. “Model 1" performed better for small-set and “Model 2" for large-set. The best value for learning rate found was 1e-3. MICE-DT: The decision tree uses Gini split criterion, unlimited tree growth, minimum number of samples for node splitting is 2, and minimum number of samples in a leaf node is 1.

**Table 4:** MC-MedGAN configurations tested

	Hyper-parameter	Model 1	Model 2
Autoencoder	Code size	64	128
	Encoder hidden size	256, 128	512, 256, 128
	Decoder hidden size	256, 128	512, 256, 128
GAN	Generator hidden layers	64, 64	128, 128, 128, 128
	Discriminator hidden size	256, 128	512, 256, 128
	# of generator/discriminator steps	2/1	3/1

For both models we used batch size of 100 samples, trained the autoencoder for 100 epochs and the GAN for 500 epochs. We applied L2-regularization on the neural network weights (weight decay) with *λ*=1e-3, and temperature parameter (Gumbel-Softmax trick) *τ*=0.66. We tested learning rates of [1e-2, 1e-3, 1e-4]

## Results

We evaluated the methods described in Section ‘Methods’ on the subsets of the SEER’s research dataset. To conserve space we only discuss results for the BREAST cancer dataset. Tables and figures for LYMYLEUK and RESPIR are shown at the end of the corresponding sections. From our empirical investigations, the conclusions drawn from the breast cancer dataset can be extended to the LYMYLEUK and RESPIR datasets. Unless stated otherwise, in all the following experiments, the number of synthetic samples generated is identical to the number of samples in the real dataset: BREAST = 169,801; RESPIR = 112,698; and LYMYLEUK = 84,132.

### On the small-set

From Table 5, we observe that many methods succeeded in capturing the statistical dependence among the variables, particularly MPoM, MICE-LR, MICE-LR-DESC, and MICE-DT. Synthetic data generated by these methods produced correlation matrices nearly identical to the one computed from real data (low PCD). Data distribution difference measured by log-cluster is also low. All methods showed a high support coverage. As seen in Fig. 2, BREAST small-set variables have only a few levels dominating the existing records in the real dataset, while the remaining levels are underrepresented or even nonexistent. This level imbalance reduces the sampling space making the methods more likely to overfit and, consequently, exposes more real patient’s information.

**Table 5:** Average and std of data utility metrics computed on BREAST small-set

	PCD *↓*	Log-Cluster *↓*	CrCl-RS (1)	CrCl-SR (1)	Supp. Coverage *↑*
Method					
IM	0.73 (0.0)	-5.38 (0.87)	0.96 (0.0)	1.0 (0.0)	0.99 (0.01)
BN	0.28 (0.01)	-8.38 (1.12)	0.99 (0.0)	1.0 (0.0)	0.98 (0.01)
MPoM	0.03 (0.01)	-10.5 (0.46)	1.0 (0.0)	1.0 (0.0)	1.0 (0.01)
CLGP	0.17 (0.01)	-7.8 (0.65)	0.99 (0.01)	1.0 (0.01)	1.0 (0.0)
MC-MedGAN	0.76 (0.01)	-3.17 (0.1)	1.0 (0.01)	0.75 (0.0)	0.95 (0.01)
MICE-LR	0.07 (0.01)	-8.34 (0.29)	0.99 (0.0)	1.0 (0.0)	1.0 (0.0)
MICE-LR-DESC	0.06 (0.01)	-9.36 (0.49)	0.99 (0.0)	1.0 (0.0)	1.0 (0.01)
MICE-DT	0.02 (0.0)	-11.61 (0.3)	1.01 (0.0)	1.0 (0.0)	0.99 (0.01)

CrCl-RS and CrCl-SR are the cross-classification metric computed on real → synthetic (RS) and synthetic → real (SR), respectively. Metrics were computed from 10 synthetically generated datasets. The symbols on the right side of metric’s name indicate: *↑* the higher the better, *↓* the lower the better, and (1) the closer to one the better

**Fig. 3:**
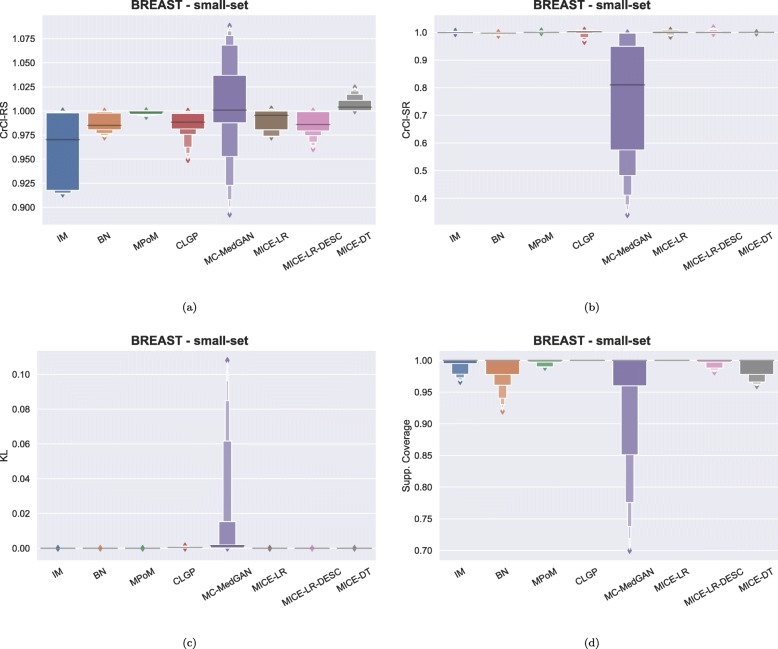
Data utility performance over all variables presented as boxplots on BREAST small-set. **a** CrCl-RS, **b** CrCl-SR, **c** KL divergence for each attribute, and **d** support coverage

**Fig. 4:**
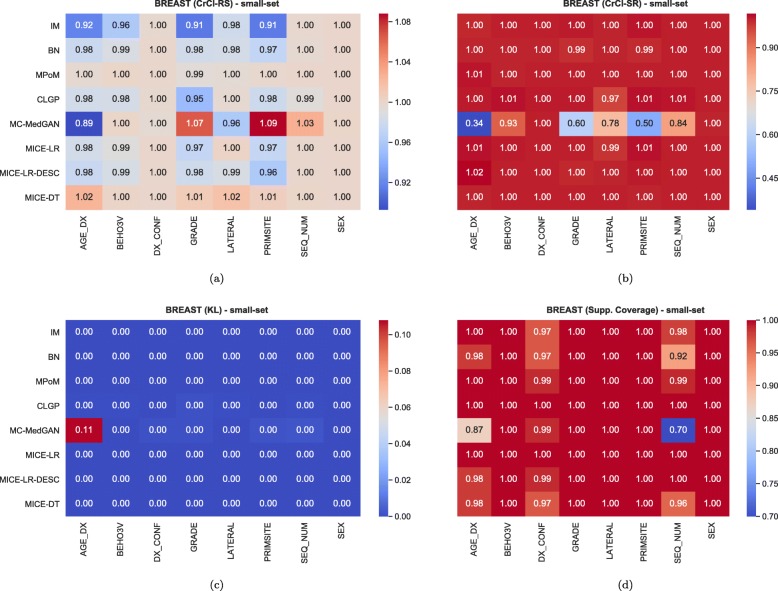
Heatmaps displaying CrCl-RS, CrCl-SR, KL divergence, and support coverage average computed over 10 independently generated synthetic BREAST small-set

Figure 3 shows the distribution of some of the utility metrics for all variables. KL divergences, shown in Fig. 3c, are low for the majority of the methods, implying that the marginal distributions of real and synthetic datasets are equivalent. KL divergences for MC-MedGAN is reasonably larger compared to the other methods, particularly due to the variable AGE_DX (Fig. 4c).

Regarding CrCl-RS in Fig. 3a, we observe that all methods are capable of learning and transferring variable dependencies from the real to the synthetic data. MPoM presented the lowest variance while MC-MedGAN has the largest, implying that MC-MedGAN is unable to capture the dependence of some of the variables. From Fig. 4a, we identify AGE_DX, PRIMSITE, and GRADE as the most challenging variables for MC-MedGAN. AGE_DX and PRIMSITE are two of the variables with the largest set of levels, with 11 and 9, respectively. It suggests that MC-MedGAN potentially faces difficulties on datasets containing variables with a large number of categories.

From Fig. 3b, we clearly note that the synthetic data generated by MC-MedGAN does not mimic variable dependencies from the real dataset, while all other methods succeeded in this task. Looking at the difference between CrCl-RS and CrCl-SR, one can infer how close the real and synthetic data distributions are. Performing well on CrCl-RS but not on CrCl-SR indicates that MC-MedGAN only generated data from a subspace of the real data distribution that can be attributed to partial modal collapse, which is a known issue for GANs [51, 52]. This hypothesis is corroborated by the support coverage value of MC-MedGAN that is the lowest among all methods.

**Fig. 5:**
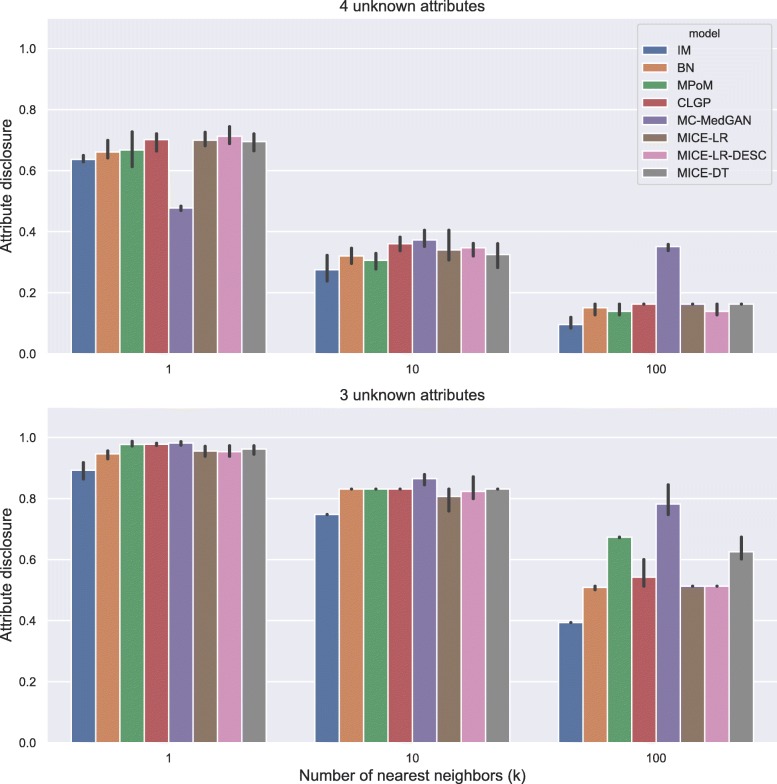
Attribute disclosure for distinct numbers of nearest neighbors (k). BREAST small-set. Top plot shows results for the scenario that an attacker tries to infer 4 unknown attributes out of 8 attributes in the dataset. Bottom plot presents the results for 3 unknown attributes

Figure 5 shows the attribute disclosure metric computed on BREAST cancer data with the small-set list of attributes, assuming the attacker tries to infer four (top) and three (bottom) unknown attributes, out of eight possible, of a given patient record. Different numbers of nearest neighbors are used to infer the unknown attributes, *k*=[1, 10, 100]. From the results, we notice that the larger the number of the nearest neighbors *k*, the lower the chance of an attacker successfully uncover the unknown attributes. Using only the closest synthetic record (*k*=1) produced a more reliable guess for the attacker. When 4 attributes are unknown by the attacker, he/she could reveal about 70% of the cases, while this rate jumps to almost 100% when 3 attributes are unknown. Notice that IM consistently produced one of the best (lowest) attribute disclosures across all cases, as it does not model the dependence across the variables. MC-MedGAN shows significantly low attribute disclosure for *k*=1 and when the attacker knows 4 attributes, but it is not consistent across other experiments with BREAST data. MC-MedGAN produced the highest value for scenarios with *k*=10 and *k*=100.

**Fig. 6:**
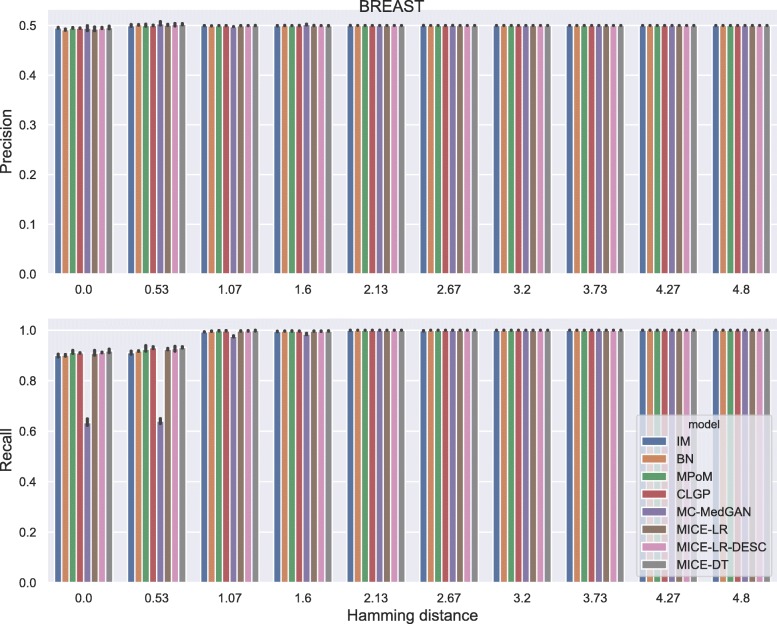
Precision and recall of membership disclosure for all methods. BREAST small-set. MC-MedGAN presents the best performance

Membership disclosure results provided in Fig. 6 for BREAST small-set shows a precision around 0.5 for all methods across the entire range of Hamming distances. This means that among the set of patient records that the attacker claimed to be in the training set, based on the attacker’s analysis of the available synthetic data, only 50% of them are actually in the training set. Regarding the recall, all the methods except MC-MedGAN showed a recall around 0.9 for the smallest prescribed Hamming distances, indicating that the attacker could identify 90% of the patient records actually used for training. MC-MedGAN presented much lower recall in these scenarios, therefore it is more effective in protecting private patient records. For larger Hamming distances, as expected, all methods obtain a recall of one as there will be a higher chance of having at least one synthetic sample within the larger neighborhood (in terms of Hamming distance). Therefore, the attacker claims that all patient records are in the training set.

Similarly to the analysis performed for the BREAST dataset, Tables 6 and 7 reports performance of the methods on LYMYLEUK and RESPIR datasets using the small-set selection of variables. Figures 7, 8, 9, 10, 11, 12, 13, and 14 present utility and privacy methods’ performance plots for the LYMYLEUK and RESPIR datasets.

**Fig. 7:**
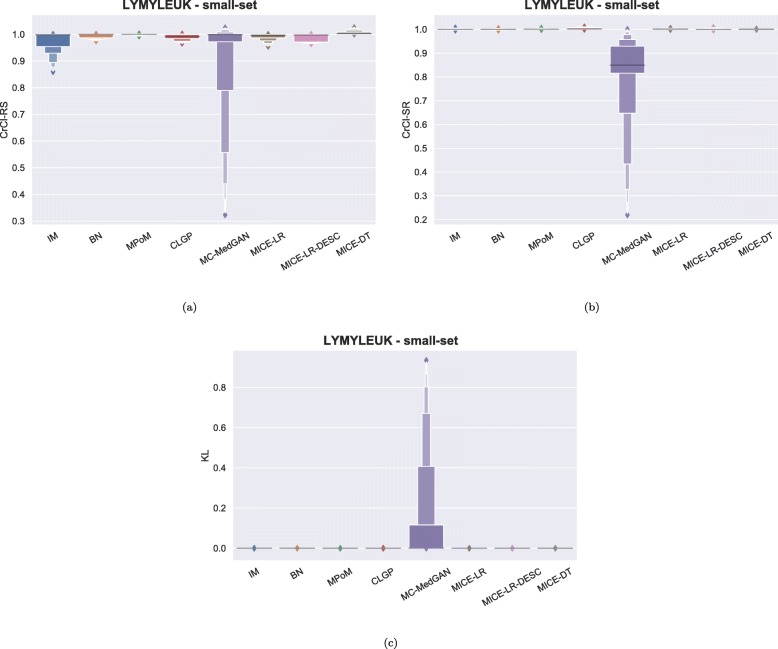
Data utility metrics performance distribution over all variables shown as boxplots on LYMYLEUK small-set

**Fig. 8:**
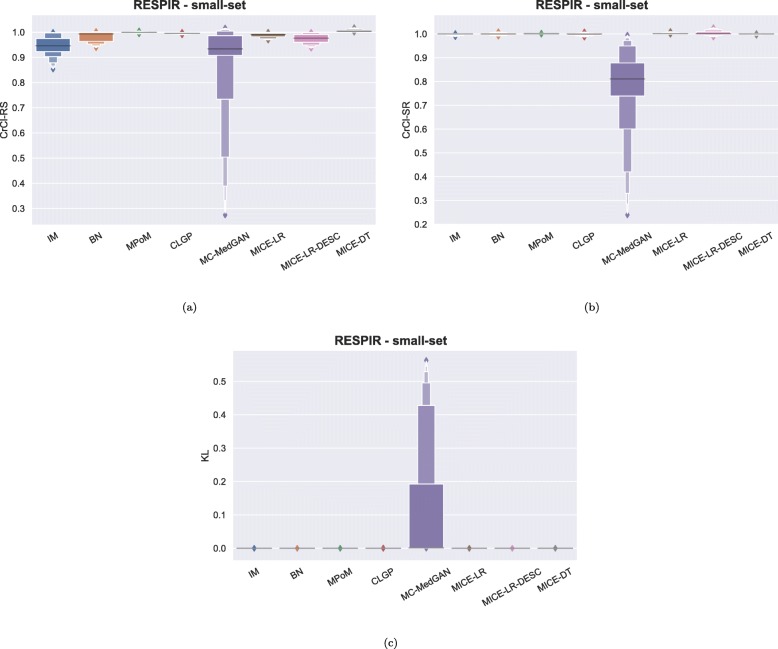
Metrics performance distribution over all variables shown as boxplots on RESPIR small-set

**Fig. 9:**
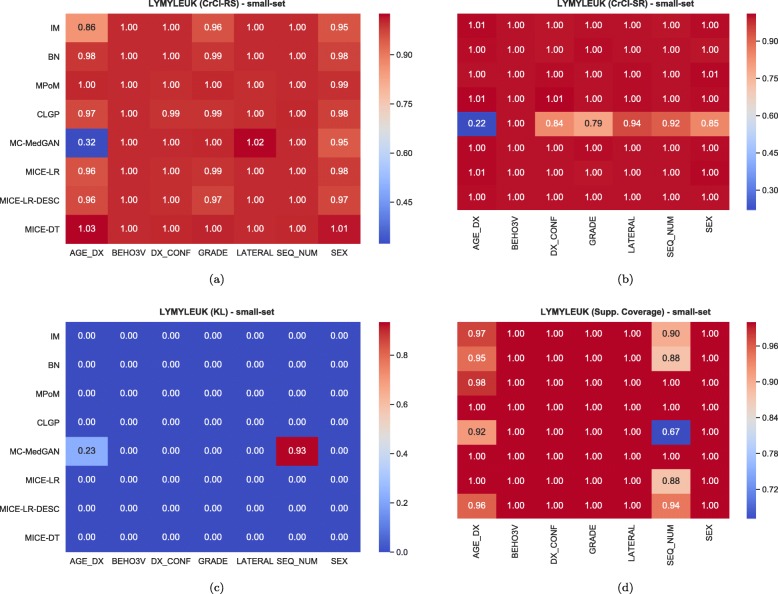
Heatmaps displaying the average over 10 independently generate synthetic datasets of (**a**) CrCl-RS, (**b**) CrCl-SR, (**c**) KL divergence, and (**d**) support coverage, at a variable level. LYMYLEUK small-set

**Fig. 10:**
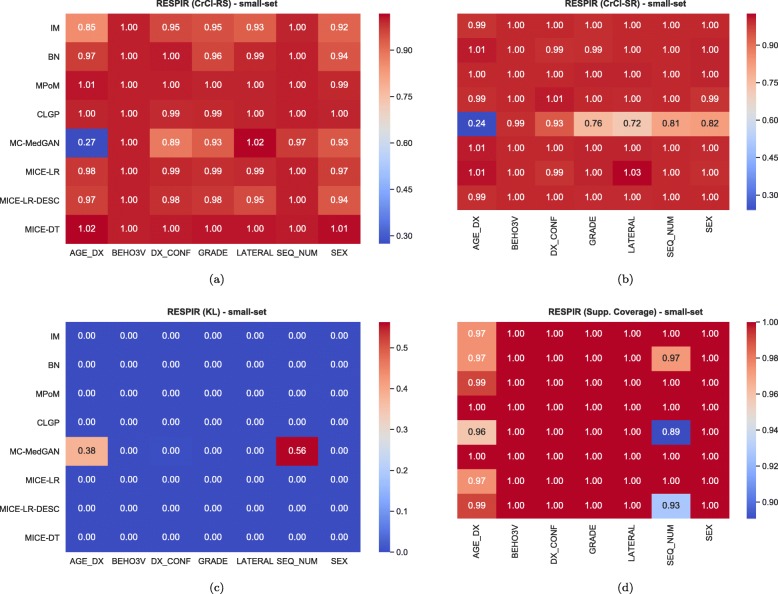
Heatmaps displaying the average over 10 independently generate synthetic datasets of (**a**) CrCl-RS, (**b**) CrCl-SR, (**c**) KL divergence, and (**d**) support coverage, at a variable level. RESPIR small-set

**Fig. 11:**
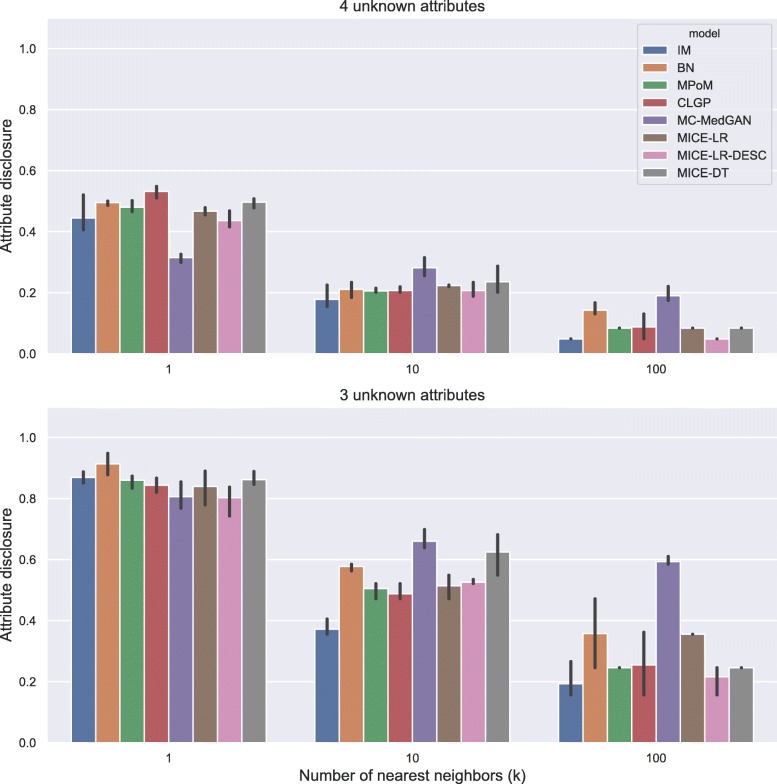
Attribute disclosure for LYMYLEUK small-set

**Fig. 12:**
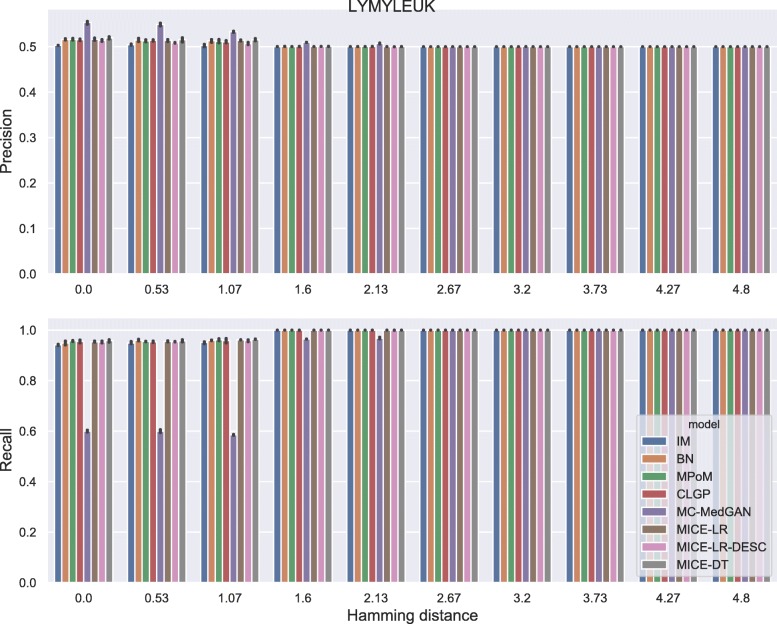
Precision and recall for membership disclosure for LYMYLEUK small-set

**Fig. 13:**
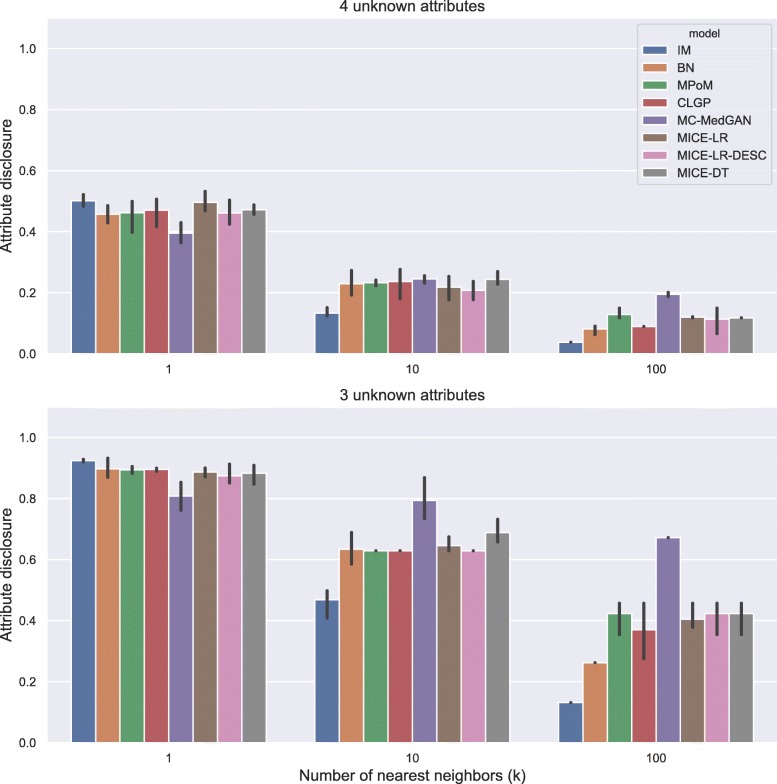
Attribute disclosure for RESPIR small-set

**Fig. 14:**
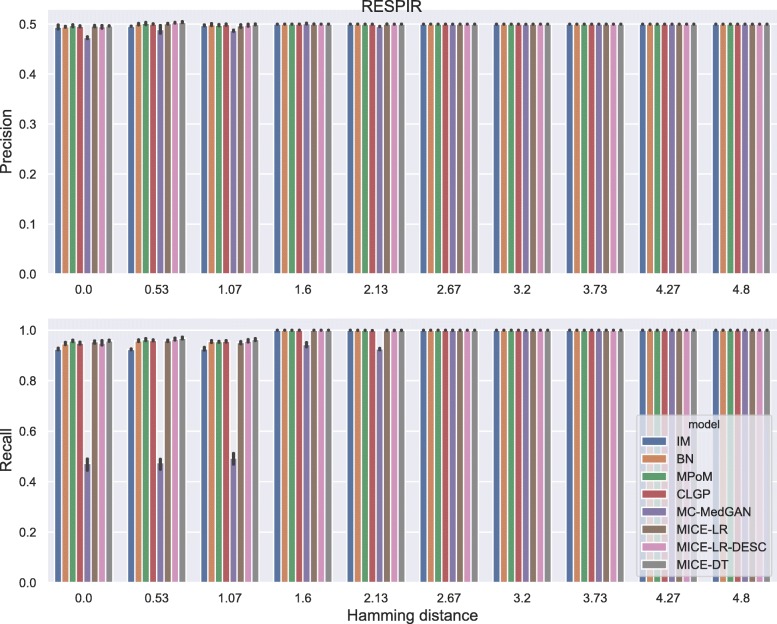
Precision and recall for membership disclosure for RESPIR small-set

**Table 6:** Average and std of data utility metrics computed over 10 independently generated synthetic datasets. LYMYLEUK small-set

	PCD *↓*	Log-Cluster *↓*	CrCl-RS (1)	CrCl-SR (1)	Supp. Coverage *↑*
Method					
IM	0.47 (0.01)	-4.94 (0.24)	0.97 (0.0)	1.0 (0.0)	0.98 (0.01)
BN	0.2 (0.01)	-6.24 (0.09)	0.99 (0.0)	1.0 (0.0)	0.98 (0.01)
PoM	0.03 (0.01)	-9.78 (0.57)	1.0 (0.0)	1.0 (0.0)	1.0 (0.01)
CLGP	0.15 (0.01)	-6.88 (1.2)	0.99 (0.01)	1.0 (0.01)	1.0 (0.0)
MC-MedGAN	0.56 (0.01)	-2.29 (0.07)	0.9 (0.01)	0.79 (0.01)	0.94 (0.01)
MICE-LR	0.04 (0.01)	-7.77 (0.36)	0.99 (0.0)	1.0 (0.0)	1.0 (0.0)
MICE-LR-DESC	0.02 (0.01)	-7.06 (0.26)	0.99 (0.0)	1.0 (0.0)	0.98 (0.01)
MICE-DT	0.02 (0.0)	-10.63 (0.32)	1.01 (0.0)	1.0 (0.0)	0.99 (0.01)

**Table 7:** Average and std of data utility metrics computed over 10 independently generated synthetic datasets. RESPIR small-set

	PCD *↓*	Log-Cluster *↓*	CrCl-RS (1)	CrCl-SR (1)	Supp. Coverage *↑*
Method					
IM	0.9 (0.0)	-3.62 (0.14)	0.94 (0.0)	1.0 (0.0)	1.0 (0.01)
BN	0.24 (0.0)	-7.47 (0.68)	0.98 (0.0)	1.0 (0.0)	0.99 (0.01)
PoM	0.03 (0.0)	-10.47 (0.37)	1.0 (0.0)	1.0 (0.0)	1.0 (0.0)
CLGP	0.13 (0.01)	-7.63 (0.52)	1.0 (0.01)	1.0 (0.01)	1.0 (0.0)
MC-MedGAN	0.64 (0.01)	-2.12 (0.06)	0.86 (0.01)	0.75 (0.01)	0.98 (0.01)
MICE-LR	0.06 (0.01)	-8.3 (0.2)	0.99 (0.0)	1.0 (0.0)	1.0 (0.0)
MICE-LR-DESC	0.04 (0.01)	-6.8 (1.2)	0.97 (0.0)	1.0 (0.0)	1.0 (0.01)
MICE-DT	0.02 (0.0)	-11.25 (0.21)	1.01 (0.0)	1.0 (0.0)	0.99 (0.01)

### On the large-set

The large-set imposes additional challenges to the synthetic data generation task, both in terms of the number of the variables and the inclusion of variables with a large number of levels. Modeling variables with too many levels requires an extended amount of training samples to properly cover all possible categories. Moreover, we noticed that in the real data a large portion of the categories are rarely observed, making the task even more challenging.

From Table 8 we observe that MICE-DT obtained significantly superior data utility performance compared to the competing models. As MICE-DT uses a flexible decision tree as the classifier, it is more likely to extract intricate attribute relationships that are consequently passed to the synthetic data. Conversely, MICE-DT is more susceptible to memorizing the private dataset (overfitting). Even though overfitting can be alleviated by changing the hyper-parameter values of the model, such as the maximum depth of the tree and the minimum number of samples at leaf nodes, this tuning process is required for each dataset which can be very time consuming. Using a MICE method with a less flexible classifier, such as MICE-LR, can be a viable alternative.

**Table 8:** Average and std of data utility metrics computed on the BREAST large-set

	PCD *↓*	Log-Cluster *↓*	CrCl-RS (1)	CrCl-SR (1)	Supp. Coverage *↑*
Method					
IM	8.94 (0.01)	-3.56 (0.12)	0.7 (0.0)	1.0 (0.0)	0.99 (0.0)
BN	4.88 (0.02)	-4.51 (0.38)	0.94 (0.0)	1.0 (0.0)	0.99 (0.0)
MPoM	2.3 (0.01)	-7.83 (0.38)	0.85 (0.0)	1.01 (0.0)	0.99 (0.0)
CLGP	0.69 (0.02)	-8.2 (0.27)	0.95 (0.0)	1.02 (0.0)	1.0 (0.0)
MC-MedGAN	– (–)	-1.39 (0.0)	0.76 (0.05)	0.58 (0.0)	0.16 (0.0)
MICE-LR	1.31 (0.04)	-5.77 (0.13)	0.87 (0.0)	1.07 (0.01)	0.99 (0.0)
MICE-LR-DESC	2.94 (0.07)	-5.09 (0.14)	0.85 (0.0)	1.04 (0.0)	1.0 (0.0)
MICE-DT	0.14 (0.02)	-11.14 (0.53)	1.01 (0.0)	1.0 (0.0)	0.99 (0.0)

CLGP presented the overall best performance. It was not possible to compute PCD metric for MC-MedGAN as the method generated at least one variable with a unique value. The symbols on the right side of metric’s name indicate: *↑* the higher the better, *↓* the lower the better, and (1) the closer to one the better

It is also worth mentioning that the order of the variables in MICE-LR has a significant impact, particularly in capturing the correlation of the variables measured by PCD. MICE-LR with ascending order produced a closer correlation matrix to the one computed in the real dataset, when compared to MICE-LR with attributes ordered in a descending manner. Our hypothesis is that by positioning the attributes with a smaller number of levels first, the initial classification problems are easier to solve and will possibly better capture the dependence among the attributes, and this improved performance will be carried over to the subsequent attributes. This is similar to the idea of curriculum learning [53].

Overall, CLGP presents the best data utility performance on the larget-set, consistently capturing dependence among variables (low PCD and CrCls close to one), and producing synthetic data that matches the distribution of the real data (low log-cluster). CLGP also has the best support coverage, meaning that all the existent categories in the real data also appear in the synthetic data. On the other extreme, MC-MedGAN was clearly unable to extract the statistical properties from the real data. As expected, IM also showed poor performance due to its lack of variables’ dependence modeling.

**Fig. 15:**
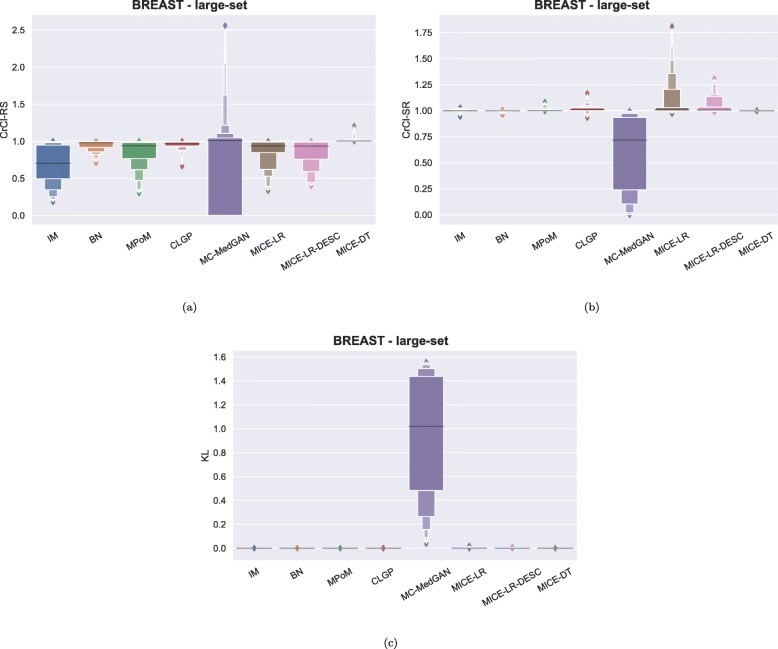
Data utility performance shown as boxplots on BREAST large-set

**Fig. 16:**
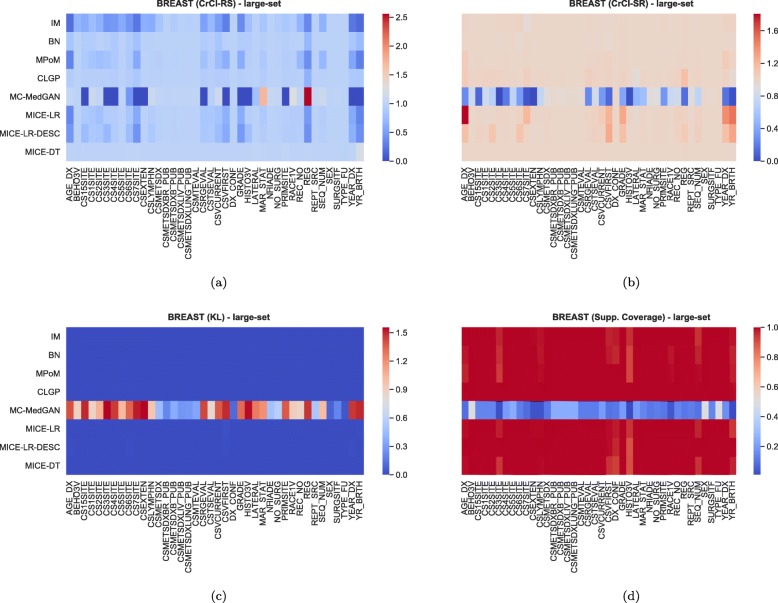
Heatmaps displaying the average over 10 independently generate synthetic datasets of (**a**) CrCl-RS, (**b**) CrCl-SR, (**c**) KL divergence, and (**d**) support coverage, at a variable level on BREAST large-set

As observed in the small-set variable selection, MC-MedGAN performed poorly on CrCl-SR metric compared to CrCl-RS (Fig. 15) and only covered a small part of the variables’ support in the real dataset. From Fig. 16a and b we note that a subset of variables are responsible for MC-MedGAN’s poor performance on CrCl-SR and CrCl-RS. Figure 16b also indicates that MICE-LR-based generators struggled to properly generate synthetic data for some variables. We also highlight the surprisingly good results obtained by BN on CrCl-RS and CrCl-SR metrics, considering the fact that BN approximates the joint distribution using a simple first-order dependency tree.

**Fig. 17:**
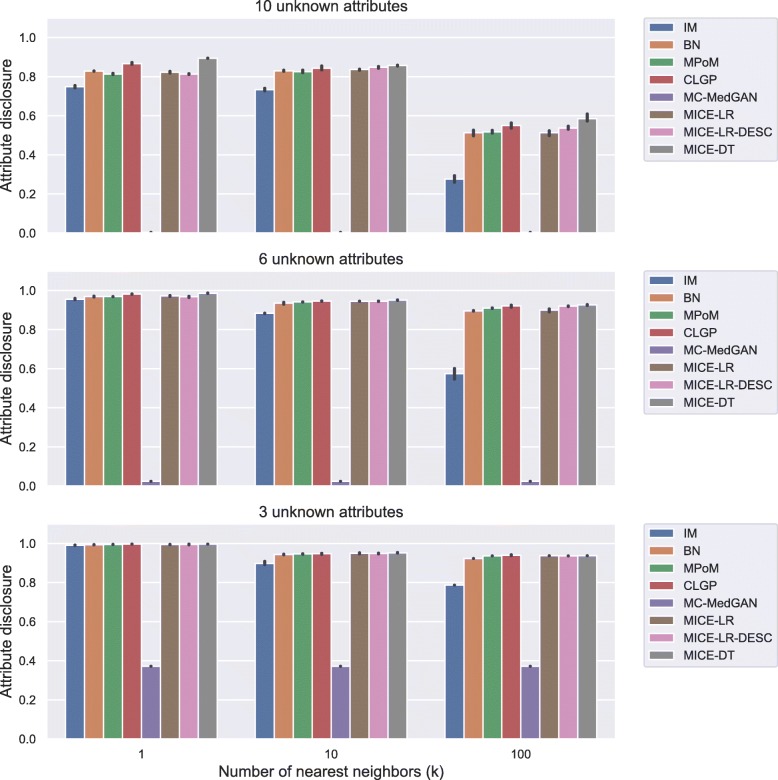
Attribute disclosure for several values of nearest neighbors (k). BREAST large-set. Results show attribute disclosure for the case an attacker seeks to infer 10, 6, and 3 unknown attributes, assuming she/he has access to the remaining attributes in the dataset

Figure 17 shows the attribute disclosure for the BREAST large-set dataset for several numbers of nearest neighbors (*k*) and three different scenarios: when the attacker seeks to uncover 10, 6, and 3 unknown attributes, assuming she/he has access to the remaining attributes in the dataset. Overall, all methods but MC-MedGAN revealed almost 100% of the cases for values of *k*=1, when 3 attributes are unknown, but decrease to about 50% when 10 attributes are unknown. Clearly, MC-MedGAN has the best attribute disclosure as a low percentage of the unknown attributes of the real records are revealed. However, MC-MedGAN produces synthetic data with poor data utility performance, indicating that the synthetically generated data does not carry the statistical properties of the real dataset. MC-MedGAN relies on continuous embeddings of categorical data obtained via an autoencoder. We believe that the complexity and noisiness of the SEER data makes learning continuous embeddings of the categorical variables (while preserving their statistical relationships) very difficult. In fact, recent work [54] has shown that autoencoders can induce a barrier to the learning process, as the GAN will completely rely on the embeddings learned by the autoencoder. Additionally, works such as [55] have reported that while GANs often produce high quality synthetic data (for example realistic looking synthetic images), with respect to utility metrics such as classification accuracy they often underperform compared to likelihood based models. IM has the second best attribute disclosure, more pronounced for *k*>1, but as already seen, also fails to capture the variables’ dependencies. The best data utility performing methods (MICE-DT, MPoM, and CLGP) present a high attribute disclosure.

**Fig. 18:**
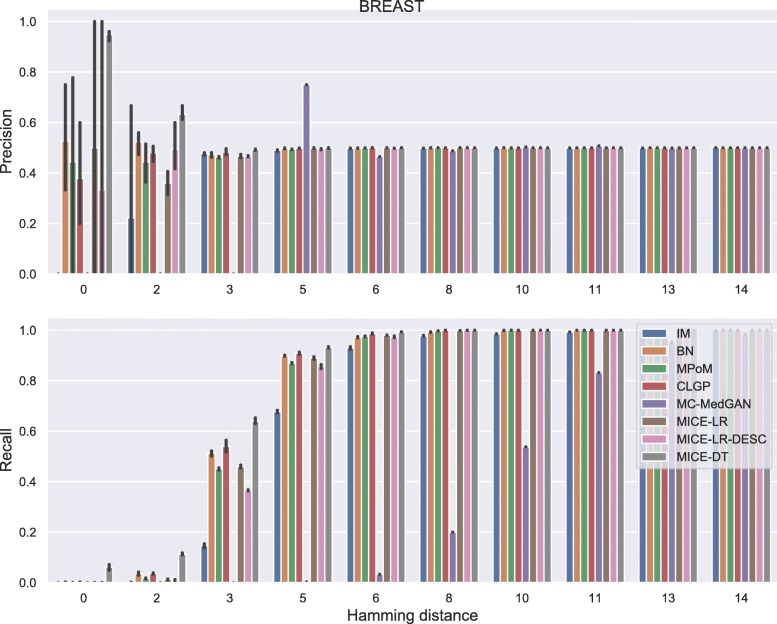
Precision and recall of membership disclosure for all methods varying the Hamming distance threshold. BREAST large-set

For membership disclosure, Fig. 18, we notice that for exact match (Hamming distance 0), some of the methods have a high membership disclosure precision, indicating that from the set of patient records an attacker claimed to be present in the training set, a high percentage of them (around 90% for MICE-DT) were correct (high precision). However, there were many true records that the attacker inferred as negative (false negative). This can be seen by the lower recall values. A conservative attacker can be successful here for MICE-DT’s synthetic dataset. As discussed previously, MICE-DT is a more flexible model that provides a high data utility performance, but is more prone to release private information in the synthetic dataset. For Hamming distances larger than 6, the attacker claims true for all patient records, as the Hamming distance is large enough to always have at least one synthetic sample within the distance threshold. It is worth mentioning that it is hard for an attacker to easily identify the optimal Hamming distance to be used to maximize its utility, except if the attacker has a priori access to two sets of patients records, one of which is present in the training set and the other is absent from the training set.

Tables 9 and 10 report performance of the methods on LYMYLEUK and RESPIR datasets using the large-set selection of variables. Figures 19, 20, 21, 22, 23, 24, 25, and 26 present utility and privacy methods’ performance plots for the LYMYLEUK and RESPIR large-set datasets.

**Fig. 19:**
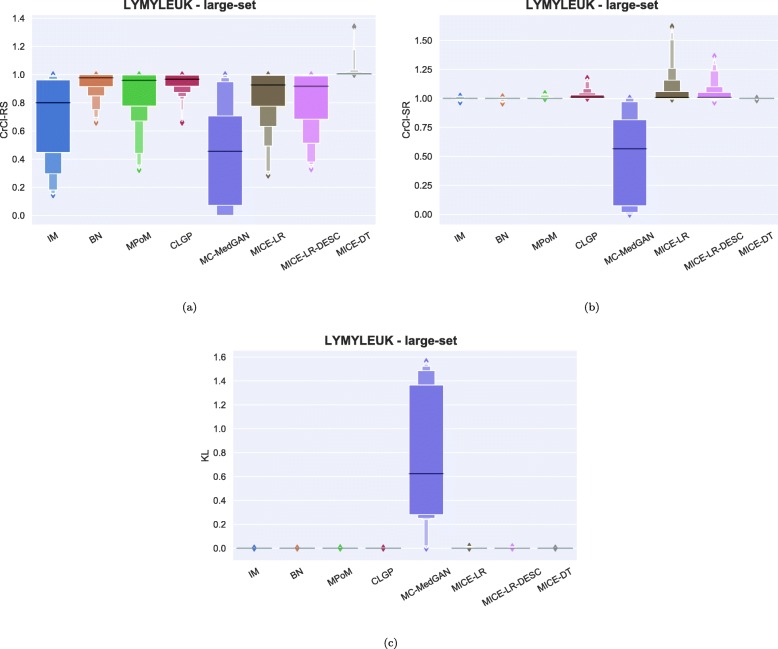
Data utility metrics shown as boxplots on LYMYLEUK large-set

**Fig. 20:**
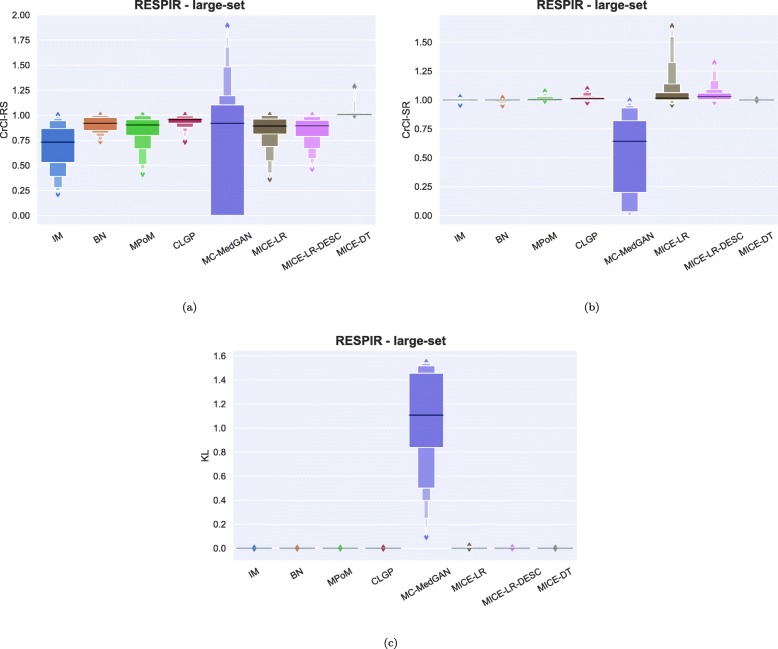
Data utility metrics shown as boxplots on RESPIR large-set

**Fig. 21:**
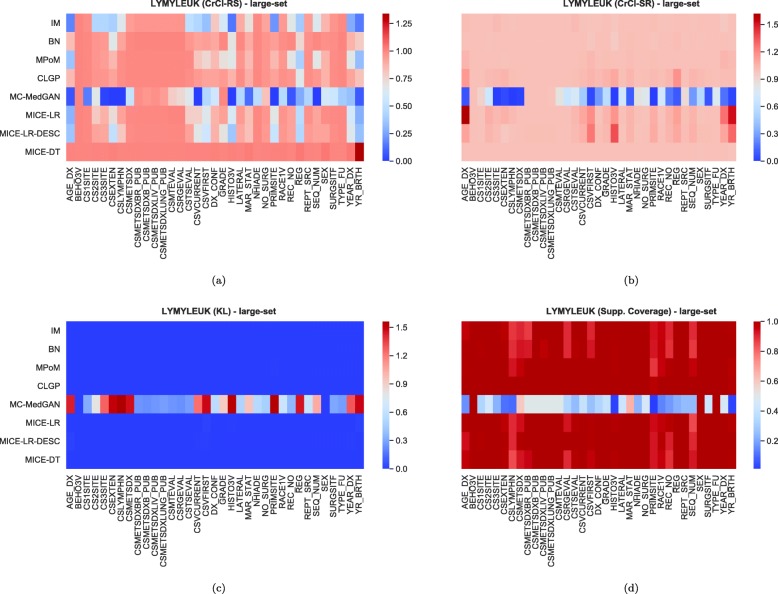
Heatmaps displaying (**a**) CrCl-RS, (**b**) CrCl-SR, (**c**) KL divergence, and (**d**) support coverage average over 10 independently generated synthetic datasets. LYMYLEUK large-set

**Fig. 22:**
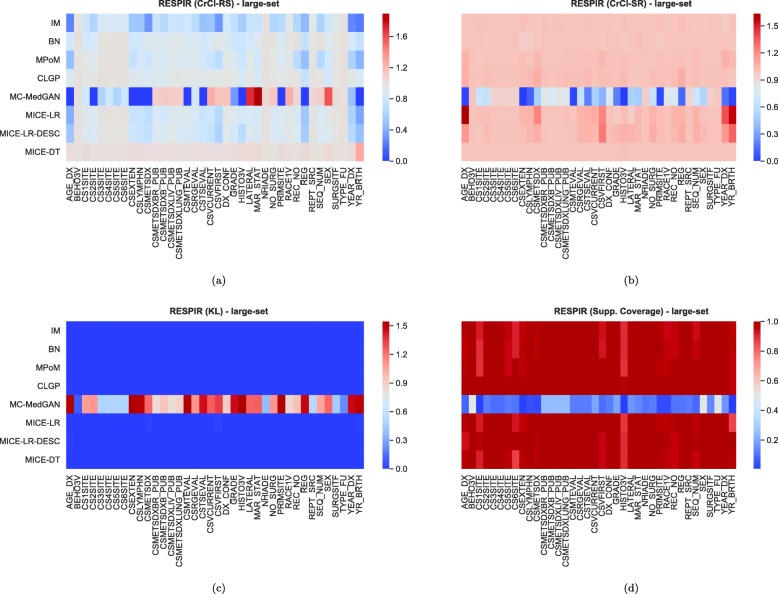
Heatmaps displaying (**a**) CrCl-RS, (**b**) CrCl-SR, (**c**) KL divergence, and (**d**) support coverage average over 10 independently generated synthetic datasets. RESPIR large-set

**Fig. 23:**
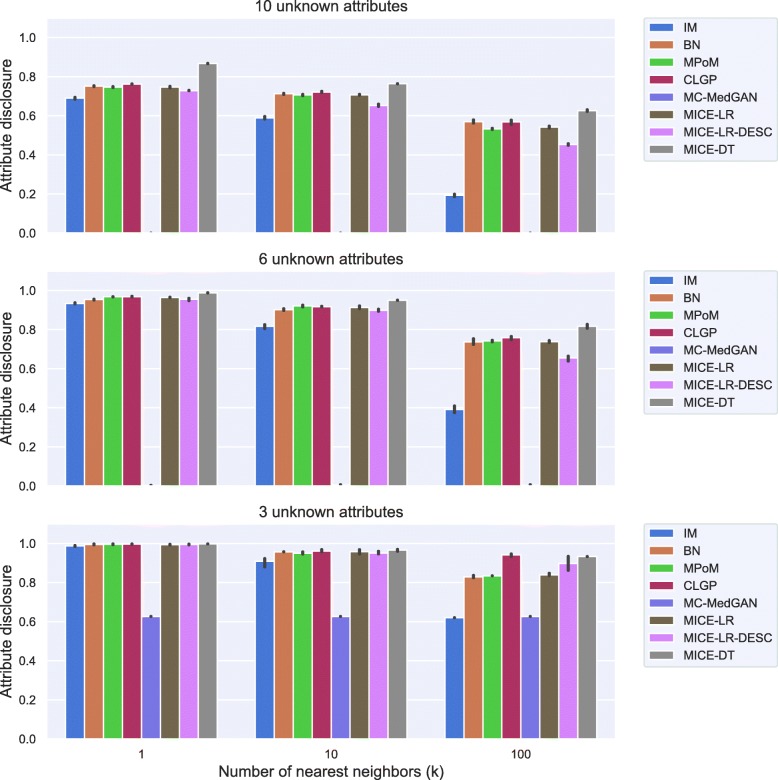
Attribute disclosure for LYMYLEUK large-set for the case 10, 6, and 3 attributes are unknown to the attacker

**Fig. 24:**
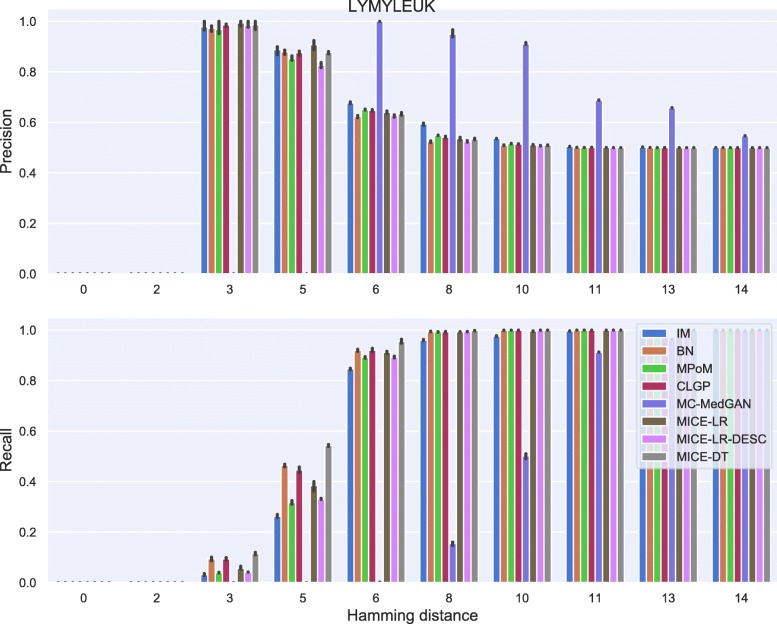
Precision and recall for membership disclosure for LYMYLEUK large-set

**Fig. 25:**
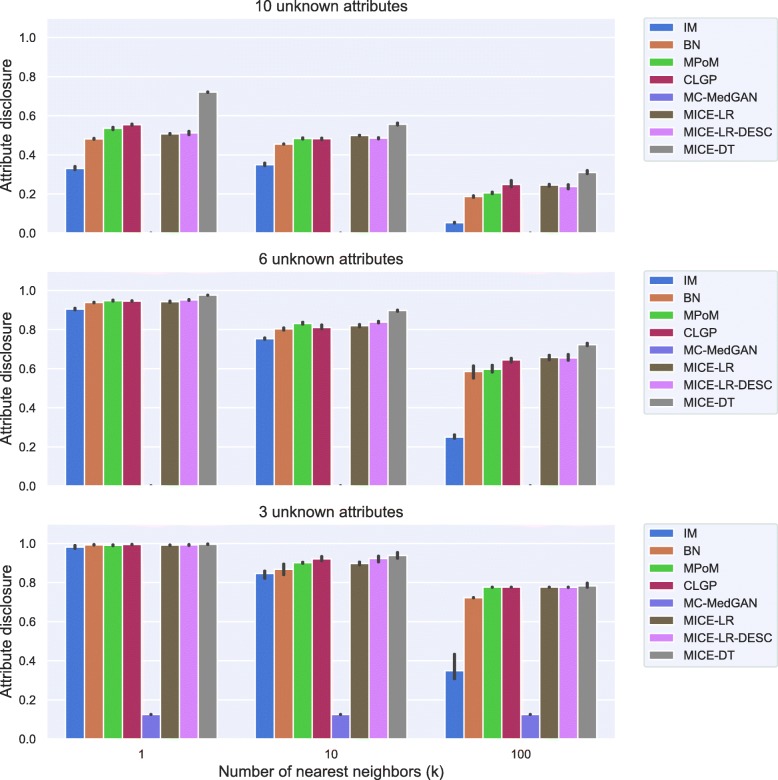
Attribute disclosure for RESPIR large-set for the case 10, 6, and 3 attributes are unknown to the attacker

**Fig. 26:**
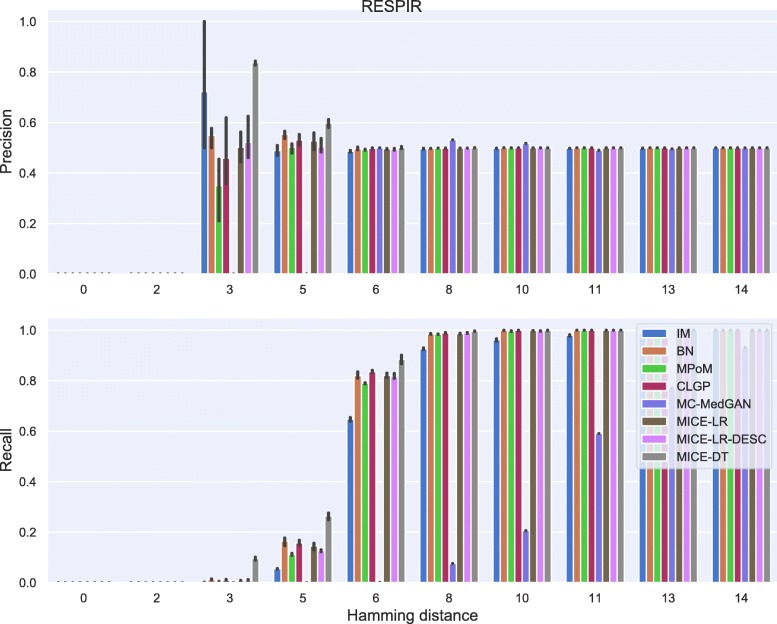
Precision and recall for membership disclosure for RESPIR large-set

**Table 9:** Average and std of performance metrics computed on 10 synthetic datasets generated from LYMYLEUK large-set

	PCD *↓*	Log-Cluster *↓*	CrCl-RS (1)	CrCl-SR (1)	Supp. Coverage *↑*
Method					
IM	9.09 (0.01)	-2.89 (0.08)	0.69 (0.0)	1.0 (0.01)	0.97 (0.01)
BN	1.59 (0.01)	-9.1 (1.08)	0.94 (0.0)	1.0 (0.0)	0.98 (0.01)
PoM	1.45 (0.01)	-7.58 (0.42)	0.86 (0.0)	1.0 (0.01)	0.99 (0.0)
CLGP	0.73 (0.02)	-8.34 (0.94)	0.94 (0.01)	1.02 (0.01)	1.0 (0.0)
MC-MedGAN	22.9 (0.02)	-1.42 (0.01)	0.45 (0.02)	0.47 (0.01)	0.38 (0.01)
MICE-LR	1.32 (0.02)	-4.8 (0.16)	0.85 (0.0)	1.07 (0.0)	0.98 (0.0)
MICE-LR-DESC	2.38 (0.04)	-3.55 (0.19)	0.82 (0.0)	1.05 (0.0)	0.99 (0.0)
MICE-DT	0.12 (0.02)	-10.92 (0.33)	1.02 (0.0)	1.0 (0.0)	0.98 (0.01)

CLGP presented the overall best performance

**Table 10:** Average and std of performance metrics computed on 10 synthetic datasets generated from RESPIR large-set

	PCD *↓*	Log-Cluster *↓*	CrCl-RS (1)	CrCl-SR (1)	Supp. Coverage *↑*
Method					
IM	9.35 (0.0)	-3.85 (0.1)	0.68 (0.0)	1.0 (0.0)	0.98 (0.0)
BN	4.59 (0.02)	-4.13 (0.14)	0.91 (0.0)	1.0 (0.01)	0.98 (0.0)
PoM	1.66 (0.01)	-6.56 (0.25)	0.85 (0.0)	1.01 (0.0)	0.99 (0.0)
CLGP	0.71 (0.02)	-7.67 (0.56)	0.94 (0.0)	1.02 (0.0)	1.0 (0.0)
MC-MedGAN	- (-)	-1.39 (0.0)	0.74 (0.03)	0.56 (0.0)	0.15 (0.0)
MICE-LR	1.35 (0.03)	-6.38 (0.15)	0.85 (0.0)	1.07 (0.0)	0.99 (0.0)
MICE-LR-DESC	2.9 (0.02)	-7.15 (0.23)	0.85 (0.0)	1.05 (0.0)	0.99 (0.0)
MICE-DT	0.13 (0.01)	-10.79 (0.78)	1.02 (0.0)	1.0 (0.0)	0.98 (0.0)

CLGP presented the overall best performance

### Effect of varying synthetic data sample sizes on the evaluation metrics

The size of the synthetic dataset has an impact on the evaluation metrics, especially on the privacy metrics. For example, a membership attack may be more difficult if only a small synthetic sample size is provided. To assess the impact of the synthetic data sample size on the evaluation metrics, we performed experiments with different sample sizes of BREAST simulated data: 5,000; 10,000; and 20,000 samples. As a reference, the results provided so far have considered a synthetic sample dataset of the same size as the real dataset, which is approximately 170,000 samples for BREAST.

Table 11 presents the log-cluster, attribute disclosure, and membership disclosure performance metrics for varying sizes of synthetic BREAST small-set datasets. We observe an improvement (reduction) of the log-cluster performance with an increase in the size of the synthetic data. A significant reduction is seen for MPoM, BN, and all MICE variations. This is likely due to the fact that with an increase in the size of the synthetic dataset, a better estimate of the synthetic data distribution is obtained. Models with lower utility metrics, such as IM and MC-MedGAN, do not show large differences in performance over the range of 5,000 to 170,000 synthetic samples. Similar behavior to log-cluster was also observed for the other utility metrics, which are omitted for the sake of brevity.

**Table 11:** LogCluster performance metric on several synthetic sample sizes

	# of synthetic samples			
	5k	10k	20k	∼170k (all)
IM	-5.072	-4.978	-5.118	-6.000
BN	-6.747	-7.053	-7.533	-8.528
MPoM	-7.106	-7.991	-8.739	-10.191
CLGP	-6.991	-7.183	-7.360	-8.056
MC-MedGAN	-3.177	-3.170	-3.238	-3.185
MICE-LR	-6.924	-7.275	-7.797	-8.323
MICE-LR-DESC	-7.232	-7.875	-8.422	-9.440
MICE-DT	-7.141	-7.867	-8.863	-11.603

BREAST small-set. Values shown are the average performance over 10 independently generated synthetic samples

The impact of sample size on the privacy metrics on the BREAST small-set are shown in Tables 12, and 13. For attribute disclosure (Table 11), we note that for the majority of the models a smaller impact on the privacy metric is observed when a larger *k* (number of nearest samples) is selected. For *k*=1, flexible models such as BN, MPoM and all MICE variations show a more than 10% increase in attribute disclosure over the range of 5000 to 170,000 synthetic samples. CLGP is more robust to the sample size, increasing only by 3%. In terms of membership disclosure (Table 13), precision is not affected by the synthetic sample size, while recall increases as more data is available. All models show an increase of 10% in recall over the range of 5,000 to 170,000 samples. This increase can be attributed to the higher probability of observing a similar real patient to a synthetic patient, as more patient samples are drawn from the synthetic data model.

**Table 12:** Attribute disclosure on several synthetic sample sizes

	# of synthetic samples							
	5k		10k		20k		∼170k (all)	
	k=1	k=10	k=1	k=10	k=1	k=10	k=1	k=10
IM	0.591	0.255	0.642	0.239	0.625	0.284	0.669	0.325
BN	0.590	0.333	0.646	0.301	0.671	0.332	0.701	0.338
MPoM	0.621	0.314	0.658	0.310	0.688	0.311	0.730	0.350
CLGP	0.666	0.305	0.674	0.313	0.685	0.324	0.693	0.350
MC-MedGAN	0.518	0.341	0.531	0.391	0.514	0.392	0.575	0.443
MICE-LR	0.633	0.320	0.635	0.320	0.678	0.306	0.741	0.314
MICE-LR-DESC	0.620	0.314	0.663	0.320	0.705	0.358	0.721	0.323
MICE-DT	0.607	0.319	0.653	0.333	0.654	0.359	0.710	0.379

BREAST small-set. Values shown are the average performance over 10 independently generated synthetic samples

**Table 13:** Membership disclosure (*Hamming distance*=0.1, *r*=1000) on several synthetic sample sizes

	# of synthetic samples							
	5k		10k		20k		∼170k (all)	
	Precision	Recall	Precision	Recall	Precision	Recall	Precision	Recall
IM	0.491	0.842	0.493	0.905	0.493	0.927	0.497	0.97
BN	0.491	0.848	0.496	0.905	0.492	0.93	0.499	0.985
MPoM	0.492	0.872	0.496	0.906	0.498	0.942	0.499	0.99
CLGP	0.496	0.867	0.497	0.913	0.498	0.943	0.500	0.988
MC-MedGAN	0.488	0.585	0.486	0.627	0.482	0.651	0.491	0.751
MICE-LR	0.497	0.868	0.497	0.909	0.500	0.957	0.499	0.988
MICE-LR-DESC	0.489	0.854	0.496	0.909	0.494	0.94	0.500	0.991
MICE-DT	0.496	0.86	0.498	0.924	0.499	0.952	0.502	0.994

BREAST small-set. Values shown are the average performance over 10 independently generated synthetic samples

We also ran similar experiments for the large-set with 40 attributes. The results are shown in Tables 14, 15, and 16. Similar conclusions as those drawn for the small-set may be drawn for the large-set.

**Table 14:** Breast large-set. Log-cluster performance metric

	# of synthetic samples			
	5k	10k	20k	∼170k (all)
IM	-3.524	-3.583	-3.553	-3.550
BN	-4.416	-4.489	-4.658	-4.539
MPoM	-6.675	-7.132	-7.515	-7.753
CLGP	-7.240	-7.632	-8.047	-8.235
MC-MedGAN	-1.388	-1.388	-1.388	-1.388
MICE-LR	-5.577	-5.605	-5.735	-5.862
MICE-LR-DESC	-4.939	-5.009	-5.083	-5.173
MICE-DT	-7.036	-7.713	-8.512	-10.593

**Table 15:** Attribute disclosure performance metric when the attacker wants for uncover 5 unknown attributes. BREAST large-set

	# of synthetic samples							
	5k		10k		20k		∼170k (all)	
	k=1	k=10	k=1	k=10	k=1	k=10	k=1	k=10
IM	0.931	0.876	0.946	0.886	0.955	0.887	0.971	0.883
BN	0.947	0.932	0.954	0.942	0.967	0.945	0.982	0.946
MPoM	0.952	0.945	0.964	0.950	0.967	0.949	0.981	0.951
CLGP	0.958	0.950	0.968	0.949	0.974	0.950	0.987	0.953
MC-MedGAN	0.024	0.024	0.024	0.024	0.024	0.024	0.024	0.024
MICE-LR	0.948	0.936	0.960	0.944	0.966	0.948	0.986	0.959
MICE-LR-DESC	0.954	0.946	0.963	0.946	0.967	0.950	0.984	0.955
MICE-DT	0.959	0.952	0.973	0.954	0.983	0.957	0.998	0.972

**Table 16:** Membership disclosure (*Hamming distance*=0.2, *r*=1000) on several synthetic sample sizes

	# of synthetic samples							
	5k		10k		20k		∼170k (all)	
	Precision	Recall	Precision	Recall	Precision	Recall	Precision	Recall
IM	0.488	0.599	0.496	0.680	0.496	0.745	0.497	0.867
BN	0.497	0.863	0.496	0.918	0.497	0.918	0.498	0.966
MPoM	0.492	0.826	0.491	0.864	0.492	0.891	0.497	0.945
CLGP	0.492	0.880	0.493	0.903	0.498	0.937	0.500	0.981
MC-MedGAN	1.000	0.002	1.000	0.002	1.000	0.002	1.000	0.002
MICE-LR	0.491	0.842	0.497	0.884	0.493	0.912	0.498	0.964
MICE-LR-DESC	0.493	0.809	0.496	0.857	0.492	0.884	0.496	0.955
MICE-DT	0.496	0.899	0.498	0.936	0.499	0.955	0.502	0.996

BREAST large-set. Values shown are the average performance over 10 independently generated synthetic samples

### Running time and computational complexity

**Fig. 27:**
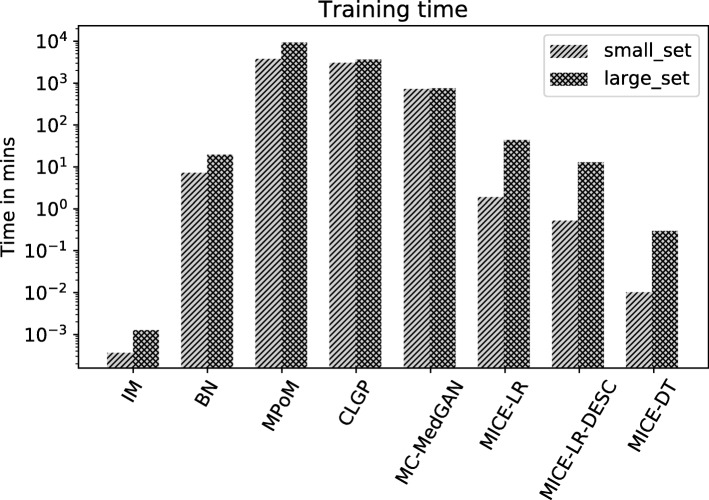
Training time in minutes for all methods on BREAST dataset considering both small-set and large-set

Figure 27 shows the training time for each method on the small-set and large-set of variables. For the range of models evaluated in this paper, the training times run from a few minutes to several days. This is primarily due to the diversity of the approaches and inferences considered in this paper. For MPoM, we performed fully Bayesian inference which involves running MCMC chains to obtain posterior samples, which is inherently costly. For CLGP, we performed approximate Bayesian inference (variational Bayes) which is computationally light compared to MCMC, however, inversion of the covariance matrix in Gaussian processes is the primary computational bottle-neck. The computation complexity of MC-MedGAN is primarily due to increased training time requirements for achieving convergence of the generator and the discriminator. The remaining approaches considered in this paper are primarily frequentist approaches based on optimization with no major computational bottle-necks. However, for the generation of synthetic datasets, the computational running time is not utterly important, since the models may be trained off-line on the real dataset for a considerable amount of time, and the final generated synthetic dataset can be distributed for public access. It is far more important that the synthetic dataset captures the structure and statistics of the real dataset, such that inferences obtained on the synthetic dataset closely reflects those obtained on the real dataset.

### Edit checks

The SEER program developed a validation logic, known as “edits”, to test the quality of data fields. The SEER edits are executed as part of cancer data collection processes. Edits trigger manual reviews of unusual values and conflicting data items. The SEER edits are publicly available in a Java validation engine developed by Information Management Services, Inc. (software^2^). All SEER data released to the public passes edits as well as several other quality measures.

There are approximately 1,400 SEER edits that check for inconsistencies in data items. The edit checks are basically if-then-else rules designed by data standard setters. Rules are implemented as small pieces of logic; each edit returns a Boolean value (true if the edit passes, false if it fails). For example, the edit that checks for inconsistent combinations of “Behavior" and “Diagnostic Confirmation" variables is represented as: “*If Behavior Code ICD-O-3[523] = 2 (in situ), Diagnostic Confirmation[490] must be 1,2 or 4 (microscopic confirmation)*".

Our purpose for using this software is to show that despite not explicitly encoding for these rules, they are implicit in the real data used to train the models (since that data passed these checks) and the models are able to generate data that for the most part does not conflict with these rules.

We ran the validation software on 10,000 synthetic BREAST samples and the percentage of records that failed in at least one of the 1400 edit checks are presented in Table 17. All methods showed less than 1% of failures on the 10 variables set. As expected, IM has the largest number of failures, as it does not take variables dependence into account when sampling synthetic data. MICE-DT, MPoM, and BN performed best. On the larger set, 40 variables, MC-MedGAN and MICE-DT show less than 1% of failures. However, as previously discussed, these two methods provided samples with high disclosure probability, and also MC-MedGAN failed to capture statistical properties of the data. We also observe that BN presented less than 2% of failed samples. Results for LYMYLEUK and RESPIR are not presented in the paper, as some information required by the validation software is not available in the public (research) version of the SEER data.

**Table 17:** Percentage of SEER edit check failures: BREAST cancer with 10 and 40 variables

Method	% of Failures	
	10vars	40vars
IM	0.53 (0.08)	6.2 (0.02)
BN	0.04 (0.01)	1.95 (0.09)
MPoM	0.01 (0.02)	4.04 (0.12)
CLGP	0.28 (0.08)	3.81 (0.22)
MC-MedGAN	0.14 (0.06)	0(0)
MICE-LR	0.12 (0.03)	4.15 (0.1)
MICE-LR-DESC	0.19 (0.05)	4.66 (0.04)
MICE-DT	0.0 (0.0)	0.06 (0.03)

It is also worth mentioning that, in practice, synthetically generated cancer cases that failed to pass at least one edit check may simply be excluded from the final list of cases to be released.

## Discussion

High quality synthetic data can be a valuable resource for, among other things, accelerating research. There are two opposing facets to high quality synthetic data. On one hand, the synthetic data must capture the relationships across the various features in the real population. On the other hand, the privacy of the subjects included in the real data must not be disclosed in the synthetic data. Here, we have presented a comparative study of different methods for generating categorical synthetic data, evaluating each of them under a variety of metrics that assess both aspects described above: data utility and privacy disclosure. Each metric evaluates a slightly different aspect of the data utility or disclosure. While there is some redundancy among them, we believe that in combination, they provide a more complete assessment of the quality of the synthetic data. For each method and each metric, we provided a brief discussion on their strengths and shortcomings, and hope that this discussion can be helpful in guiding researchers in identifying the most suitable approach for generating synthetic data for their specific application.

The experimental analysis was performed on data from the SEER research database on 1) breast, 2) lymphoma and leukemia, and 3) respiratory cancer cases diagnosed from 2010 to 2015. Additionally, we performed the same experiments on two sets of categorical variables in order to compare the methods under two challenge levels. Specifically, in the first set, 8 variables were included such that the maximum number of levels (i.e., number of unique possible values for the feature) was limited to 14. The larger feature set encompassed 40 features, including features with up to over 200 levels. Increasing the number of features and the number of levels per features results in a substantially larger parameter space to infer, which is aggravated by the absence or limited number of samples representing each possible combination.

From the experimental results on the two datasets of distinct complexity, small-set and large-set, we highlight the key differences:

- The small-set records have fewer and less complex variables (in terms of the number of sub-categories per variable) than the large-set. Thus the learning problem is considerably easier and this is observed in the metric CrCl-RS provided in Tables 5 and 8, where the small-set performs consistently better than the large-set across all datasets (BREAST, LYMYLEUK, and RESPIR).
- SEER edit checks consist of a set of rules combined via various logical operators. For the large-set, the rules are significantly more complex and the chances of failure are higher. This is observed in Table 17, where the percentage of failure is higher for the large-set compared to the small-set, across all methods.
- As the dimensionality (as well as complexity, as some of the variables have a larger number of sub-categories) of the records in the large-set is considerably higher than the records in the small-set, in general, it is harder for an attacker to identify the real patient records used for model training. This is observed in Fig. 18, where to achieve similar recall values for the membership attacks, the Hamming neighborhood has to be considerably larger for the large-set compared to the small-set.

The results showed that Bayesian Networks, Mixture of Product of Multinomials (MPoM) and CLGP were capable of capturing variables relationships, considering the data utility metrics used for comparison. Surprisingly, the generative adversarial network-based model MC-MedGAN failed to generate data with similar statistical characteristics to the real dataset.

## Conclusions

In this paper, we presented a thorough comparison of existing methodologies to generate synthetic electronic health records (EHR). For each method, the process is as follows: given a set of private and real EHR samples, fit a model, and then generate new synthetic EHR samples from the learned model. By learning from real EHR samples, it is expected that the model is capable of extracting relevant statistical properties of the data.

From the performed experimental analysis, we observed that there is no single method that outperforms the others in all considered metrics. However, a few methods have shown the potential to be of great use in practice as they provide synthetic EHR samples with the following two characteristics: 1) statistical properties of the synthetic data are equivalent to the ones in the private real data, and 2) private information leakage from the model is not significant. In particular, we highlight the methods Mixture of Product of Multinomials (MPoM) and categorical latent Gaussian process (CLGP). Other methods, such as the Generative Adversarial Network (GAN), were not capable of generating realistic EHR samples.

Future research directions include handling variable types other than categorical, specifically continuous and ordinal. A more in-depth investigation of the limitations of GANs for medical synthetic data generation is also required.

## Data Availability

The SEER data is publicly available, and can be requested at https://seer.cancer.gov/data/access.html. Python code for the methods and metrics described here will be made available upon request.

## Abbreviations

BN: Bayesian network
CLGP: Categorical latent Gaussian process
CrCl: Cross classification
DNN: Deep neural networks
EHR: Electronic health records
GAN: Generative adversarial network
IM: Independent marginals
KL: Kullback-Liebler
MICE: Multiple imputation by chained equations
ML: Machine learning
MPoM: Mixture of product of multinomials
PCD: Pairwise correlation difference
SDC: Statistical disclosure control
SDL: Statistical disclosure limitation
SEER: Surveillance, epidemiology, and end results program

## Footnotes

1 https://seer.cancer.gov/data/

2 https://github.com/imsweb/validation
